# Do sputum or circulating blood samples reflect the pulmonary transcriptomic differences of COPD patients? A multi-tissue transcriptomic network META-analysis

**DOI:** 10.1186/s12931-018-0965-y

**Published:** 2019-01-08

**Authors:** Rosa Faner, Jarrett D. Morrow, Sandra Casas-Recasens, Suzanne M. Cloonan, Guillaume Noell, Alejandra López-Giraldo, Ruth Tal-Singer, Bruce E. Miller, Edwin K. Silverman, Alvar Agustí, Craig P. Hersh

**Affiliations:** 1 Centro de Investigación Biomédica en Red de Enfermedades Respiratorias (CIBERES), C/Casanova 143, Cellex, P2A, 08036 Barcelona, Spain; 2 Channing Division of Network Medicine, Brigham and Women’s Hospital, Boston, MA USA; 3 Division of Pulmonary and Critical Care Medicine, Joan and Sanford I. Weill Department of Medicine, Weill Cornell Medical College, New York, NY USA; 4 Respiratory Institute, Hospital Clinic, IDIBAPS, University of Barcelona, Barcelona, Spain; 5 Respiratory Therapy Area Unit GSK R and D, Collegeville, PA USA

**Keywords:** mRNA, Chronic bronchitis, Emphysema, Co-expression network analysis

## Abstract

**Background:**

Previous studies have identified lung, sputum or blood transcriptomic biomarkers associated with the severity of airflow limitation in COPD. Yet, it is not clear whether the lung pathobiology is mirrored by these surrogate tissues. The aim of this study was to explore this question.

**Methods:**

We used Weighted Gene Co-expression Network Analysis (WGCNA) to identify shared pathological mechanisms across four COPD gene-expression datasets: two sets of lung tissues (L1 *n* = 70; L2 *n* = 124), and one each of induced sputum (S; *n* = 121) and peripheral blood (B; n = 121).

**Results:**

WGCNA analysis identified twenty-one gene co-expression modules in L1. A robust module preservation between the two L datasets was observed (86%), with less preservation in S (33%) and even less in B (23%). Three modules preserved across lung tissues and sputum (not blood) were associated with the severity of airflow limitation. Ontology enrichment analysis showed that these modules included genes related to mitochondrial function, ion-homeostasis, T cells and RNA processing. These findings were largely reproduced using the consensus WGCNA network approach.

**Conclusions:**

These observations indicate that major differences in lung tissue transcriptomics in patients with COPD are poorly mirrored in sputum and are unrelated to those determined in blood, suggesting that the systemic component in COPD is independently regulated. Finally, the fact that one of the preserved modules associated with FEV1 was enriched in mitochondria-related genes supports a role for mitochondrial dysfunction in the pathobiology of COPD.

**Electronic supplementary material:**

The online version of this article (10.1186/s12931-018-0965-y) contains supplementary material, which is available to authorized users.

## Background

Chronic Obstructive Pulmonary Disease (COPD) is defined, and its severity graded, simply by the presence of persistent airflow limitation, as determined by the forced expiratory volume in 1 s (FEV1) and the ratio of FEV1 to forced vital capacity (FVC) [1]. However, COPD is a complex and heterogeneous disease, both clinically [2] and molecularly [3, 4], and often patients have both pulmonary and systemic manifestations [5]. On the other hand, since the lung parenchyma is difficult to access, the pathobiology of COPD is often studied in surrogate tissue samples, such as sputum or circulating blood. In these surrogate tissues, different mRNAs whose expression is associated with the severity of airflow limitation have been identified. [6–11] Yet, the relationship of changes in these surrogate tissues with those occurring in the lung parenchyma is unclear.

Network analysis is a novel research strategy well-suited to integrate and analyze complex data sets and to investigate complex and heterogenous diseases such as COPD [3, 12]. Weighted gene co-expression network correlation-based analysis (WGCNA) is a particular type of network analysis that allows the identification of modules of co-expressed genes in a given transcriptomic dataset, the investigation of the degree of module preservation in other datasets, and the study of their relationship with clinical features of interest [13–15]. This network based comparison can be performed using data from different technological platforms [13–15]. Here, we used WGCNA to meta-analyse four previously published COPD transcriptomic datasets determined in lung tissue (L), blood (B) and sputum (S) [3, 16–18] *(1)* to identify L modules related with FEV 1, *(2)* to investigate if these L modules are preserved in S and/or B data sets, and (3) to investigate the biological processes associated with these modules.

## Methods

Methods are detailed in the Additional file 1.

## Participants and data sets

We used four transcriptomic datasets generated by three different COPD studies in Lung (L1 [3] and L2 [16]), induced sputum (S) and circulating blood (B) [17, 18]. L1 included 70 lung tissue samples obtained from COPD patients who spanned all GOLD grades (1–4) of airflow limitation severity [3], L2 included 90 lung tissue samples from COPD patients with GOLD grades 3–4 and 34 former smokers with normal lung function. The S and B datasets were obtained from 121 COPD patients included in the ECLIPSE study with GOLD grades 2–4 [17]. To avoid a potential confounder effect of active smoking on transcriptomics, all participants were former smokers, who had been abstinent from smoking for at least one month before tissue sampling. The selected datasets fulfilled the following criteria: including former smokers with COPD (to avoid the influence of the active smoking exposure in the transcriptomic results), large sample size (n > =70), being of the same individual (blood and sputum).

## Ethics statement

The Ethic Committees of the participating institutions approved each of these three studies, and all participants provided written informed consent prior to the performance of any study procedures.

## Gene expression

The methodology for microarray hybridization has been described previously [3, 16–18]. All datasets are available on the Gene Expression Omnibus website, http://www.ncbi.nlm.nih.gov/geo/ (GSE69818, GSE4837, GSE22148 and GSE76925). The array platforms used in each cohort were: i) L1, Human Genome U219 Array Plate (Affymetrix, Santa Clara, CA, USA), ii) L2, HumanHT-12 v4 Expression BeadChip Kit (Illumina, San Diego, USA); and iii) S and B (ECLIPSE), HG_Plus_2.0 GeneChips (Affymetrix, Santa Clara, CA, USA).

## qPCR validation

In 20 additional lung tissue samples, recruited at Hospital Clinic of Barcelona (characteristics provided in the Additional file 1), the expression of MPV17L2, TSFM, and NDUFA3 was assessed by qPCR using TaqMan assays and 2^ΔCP^ with ACTB as the housekeeping gene, based on previously described methods [3].

## Data analysis

Quantitative clinical data is presented as mean ± standard deviation and compared between groups using one-way ANOVA. Microarray pre-processing of each dataset has been previously described [3, 16–18]. For the present analysis, probes in the lowest quartile of variability were removed, and array probes were collapsed to genes, yielding 10,434 genes for final analysis.

### Weighted gene co-expression network meta-analysis

The WGCNA meta-analysis was performed using the WGCNA R package [13, 14] following the previously described meta-analysis pipeline [15]. A step by step tutorial on how to perform the WGCNA package can be found at: https://horvath.genetics.ucla.edu/html/CoexpressionNetwork/Rpackages/WGCNA/Tutorials/.

Briefly, we first calculated the correlation matrix and defined the WGCNA co-expression modules (labelled by colour) in L1. The adjacency matrix for each data set was built using the biweight midcorrelation, with a softpower threshold of 12. The DeepSplit for module identification in LT-1 was 1 and the minimum module size 30. WGCNA produces a set of modules (labeled by color), each containing a set of unique genes. Next, the preservation of the modules across the other three datasets was assessed using the modulePreservation function with 100 permutations; Zsummary values > 5 were considered as preserved modules [15, 19]. The module *eigengene* value (i.e., the first principal component of the expression matrix of the probes within the module) was calculated and used to test for association with the severity of airflow limitation (as expressed by the FEV_1_% predicted) after adjusting for gender and body mass index (BMI) [20]. False discovery rate (FDR) < 0.05 was used to define association. Driver genes were identified as those with highest Module Membership (kME) in LT1 modules that also have highest kME in the same modules in the other datasets [15]. To test the association of blood gene expression with level of airflow limitation and verify the lack of association, WGCNA modules were defined for this dataset independently of the others, and their association with FEV1 was calculated.

### Enrichment analysis

To identify over-represented pathways in co-expression modules related to FEV_1_% predicted, we used hypergeometric tests in the R Bioconductor package GeneAnswers [21], the Vignettes describing how to use the pipeline are available from Bioconductor (https://www.bioconductor.org/). Significant enrichment was defined as FDR < 0.05, with five or more genes associated to the term. To evaluate the term overlap and obtain a visual summary we used REVIGO [22], a tutorial is available at: http://revigo.irb.hr/).

### Consensus network

A WGCNA consensus network was built using data from L1, L2 and S using the blockwiseConsensusModules function with a softpower of 12, minModuleSize of 30, a maxBlockSize of 10,434, a corType “bicor”, and the network and the TOM were signed [19]. Then, we used linear regression with adjustment for gender and BMI to identify modules associated with FEV_1_% predicted [20].

## Results

### Characteristics of participants

Table 1 describes the main characteristics of participants. All patients were Caucasians and former smokers. There were more women in the L2 cohort, and ECLIPSE participants had smoked fewer pack-years, but age and BMI were similar across the three studies.

**Table 1:** Characteristics of the 3 cohorts (4 transcriptomic datasets)

	L1	L2	ECLIPSE	
Sample Type and GEO accession number	Lung Tissue	Lung Tissue	Sputum & Blood	*p* value
	GSE69818	GSE76925	GSE22148, GSE76705	
Number of COPD/Controls	70/0	90 /34	121/0	
Gender (M/F)	63/7	57/67	81/40	0.0005
Age	66.3 ± 8.6	64.3 ± 7.3	65.1 ± 5.5	ns
Current/Former Smokers	0/70	0/124	0/121	ns
Pack-years of smoking	56.5 ± 26.1	57.2 ± 28.6	47.1 ± 29.2	0.012
BMI	27.5 ± 4.5	26.2 ± 4.6	26.6 ± 4.9	ns
FEV_1_% predicted	57.9 ± 21.1	46.1 ± 34.0	48.9 ± 15.1	< 0.0001
FEV_1_/FVC	52.8 ± 12.3	45.1 ± 22.8	42.6 ± 11.8	0.0075

ns: non-significant *p* value > 0.05. GEO = gene expression omnibus

### Network based transcriptomic meta-analysis

**Fig. 1:**
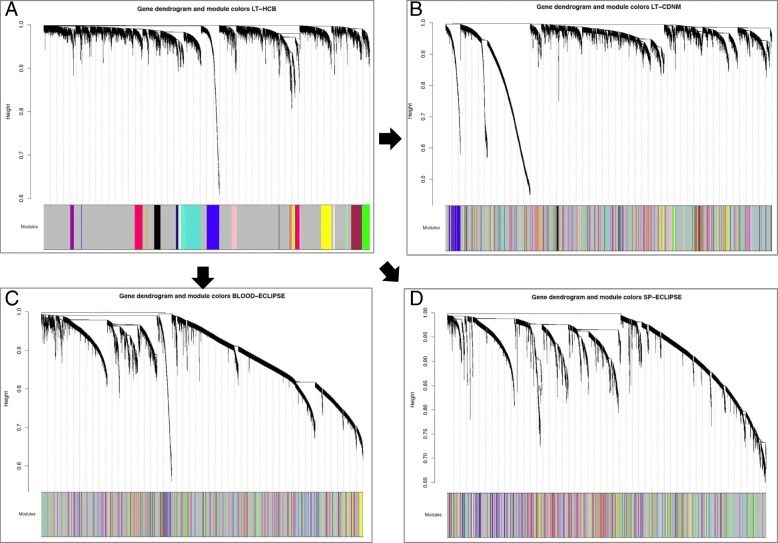
Co-expression network dendrograms in the 4 datasets. (**a**) L1 co-expression network, with twenty-one modules with a minimum module size of 30. (**b**) L2 co-expression network. (**c**) Blood co-expression network. (**d**) Sputum co-expression network. The color code in parts B, C, and D corresponds to the modules identified in L1 (part A). For further details on analytical method see: (https://horvath.genetics.ucla.edu/html/CoexpressionNetwork/Rpackages/WGCNA/Tutorials/)

Using WGCNA [13], we identified 21 modules with a minimum size of 30 genes in the L1 (Fig. 1, panel A). These modules were also identified in L2, S and B datasets (Fig. 1, panels B/C/D). Using the Z preservation score, we ranked their preservation across datasets (Table 2). Z-score values > 5 are considered preserved modules [19]. Four modules (19%) were preserved across all datasets (Table 2). Eighteen modules (86%) were preserved in the two lung tissue datasets, whereas seven (33%) were also preserved in sputum, and five (24%) were preserved in blood (Table 2). The negative control, a random selection of genes (gold module), was not preserved in any group.

**Table 2:** Module preservation across the different datasets

		L2	ECLIPSE-BL	ECLIPSE-SP
Module ID	# genes	Z score	Z score	Z score
Blue	394	**29.91**	0.41	1.35
Yellow	329	**17.02**	**10.32**	**23.76**
Brown	345	**16.28**	1.75	**8.86**
Salmon	91	**11.82**	0.00	2.07
Green yellow	99	**11.41**	2.39	1.37
Light yellow	37	**9.38**	2.58	2.31
Pink	148	**11.57**	0.91	−0.71
Light green	51	**8.65**	4.44	**6.16**
Tan	95	**10.76**	−0.47	−0.79
Magenta	142	**8.74**	2.27	**5.44**
Red	238	**8.03**	**5.61**	1.02
Black	204	**7.89**	−0.47	0.62
Turquoise	400	**6.84**	**14.23**	**9.04**
Green	241	**7.90**	1.72	3.21
Midnight blue	76	**5.81**	0.19	−0.74
Grey60	56	**7.26**	**5.38**	**5.48**
Royal blue	30	**6.30**	**9.02**	**6.43**
Cyan	82	**6.04**	1.81	0.35
Light cyan	61	4.14	1.07	0.43
Purple	113	2.71	2.58	2.00
Gold	400	2.86	3.53	2.71
Grey	400	2.76	4.09	4.89

Modules are considered preserved if Z score is > 5 (in bold and underlined in the table), and highly preserved if Z score is > 10 as described in reference [19]

### Association with lung function

**Fig. 2:**
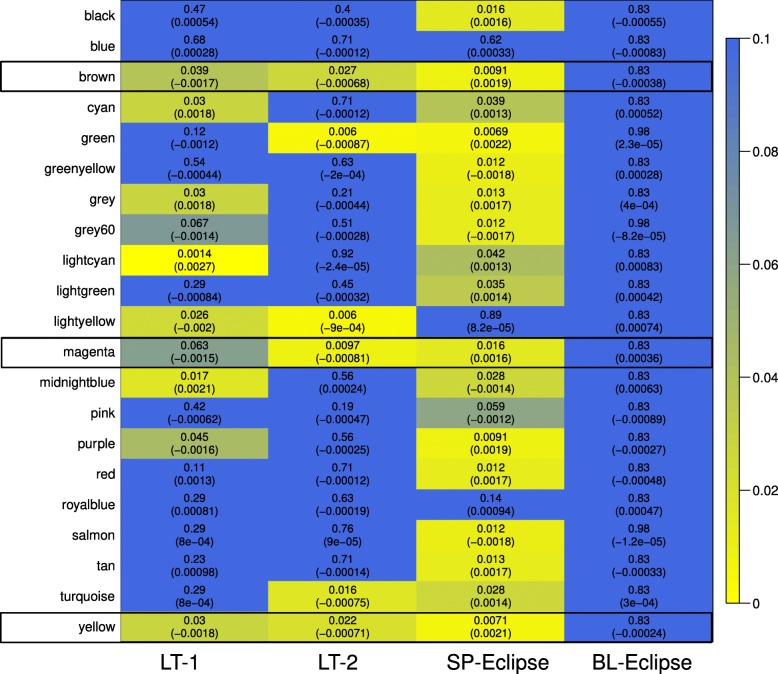
Association between meta-analysis gene modules and lung function. Heat-map shows the *p*-values (and effect estimates) of the linear regression of each module eigengene with FEV1% predicted in each of the four datasets. Yellow denotes lower p-values and blue higher p-values. The three preserved modules associated with FEV_1_% predicted are marked with a black outline. For further details on analytical method see: (https://horvath.genetics.ucla.edu/html/CoexpressionNetwork/Rpackages/WGCNA/Tutorials/)

**Fig. 3:**
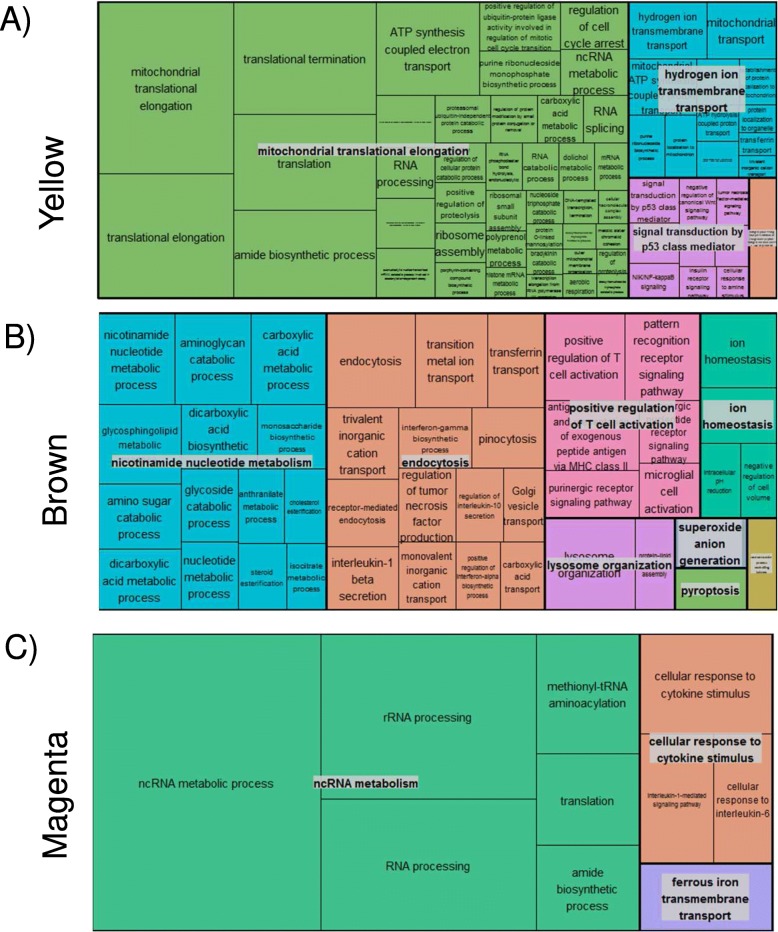
Visual summary of the ontologies in each of the three interesting modules (Treemap in REVIGO (http://revigo.irb.hr/) [22]): (**a**) Yellow, (**b**) Brown, (**c**) Magenta. Each box is a single ontology cluster, which are joined into superclusters of loosely related terms, shown by the same color and named with a representative term. Larger boxes have more significant enrichment

To investigate the relationships between identified modules and FEV1, we performed a linear regression of each module Eigengene with the FEV_1_% predicted as the dependent variable, after adjusting for covariates (gender and BMI). Results are displayed in the form of a heat map in Fig. 2 (*p*-value and effect estimate). No blood module was associated with FEV1% at *p* < 0.05, even when the module definition was done in the blood dataset, (Additional file 2: Figure S1). By contrast, 8 modules (38%) were significantly associated with airflow limitation in L1, 6 (28%) in L2, and 17 (81%) in S; 3 of them were preserved in L1, L2 and S: *yellow* (329 genes), *brown* (345 genes) and *magenta* (142 genes) modules (Fig. 2, Table 2 and Additional file 3: Table S1). The *yellow* module (Fig. 3a, Additional file 4: Table S2) included ontologies related to mitochondrial function, signal transduction by p53, hydrogen ion transmembrane transport and MHC class I antigen processing/presentation. Accordingly, KEGG pathways analysis showed enrichment in oxidative phosphorylation, metabolic and spliceosome pathways (Additional file 5: Table S3). The *brown* module (Fig. 3b, Additional file 4: Table S2) includes ontologies related to endocytosis, lysosome organization, ion homeostasis, nucleotide metabolism and T cell activation. KEGG pathway analysis showed enrichment in Lysosome and Phagosome pathways (Additional file 5: Table S3). Finally, the *magenta* module (Fig. 3c, Additional file 4: Table S2) includes ontologies related to noncoding RNA metabolism, cellular response to cytokine stimulus and iron transmembrane transport. KEGG analysis showed enrichment in the Ribosome biogenesis pathway (Additional file 5: Table S3).

We defined *driver genes* in a module as those genes both associated with the severity of airflow limitation and also highly correlated with other genes in that module in all datasets (see methods) [15]. Table 3 lists the driver genes in the three modules of interest and highlighted in bold those genes that are included the enriched gene ontology categories. In the *yellow* module, ANAPC11, ATP5G1, ATP5G2, and NDUFA13 were included in the mitochondrial related ontologies. In the *brown* module, APEH and GPI were included in the metabolism related ontologies. In the *magenta* module ABCE1, MKI67IP, RIOK1, TIMM17A and WDR43 were included in the RNA related ontologies.

**Table 3:** Driver genes in preserved modules associated with lung function meta-analysis modules in the 4 datasets

Yellow module	Brown module	Magenta module
ANAPC11	APEH	ABCE1
ATP5G1	CDK5	ATL3
ATP5G2	CHCHD10	CYCS
MRPL23	CST3	GGCT
MRPS12	DCAF7	MKI67IP
NDUFA13	GPI	MRPL32
NDUFS3	PLD3	NUP35
ROMO1	PSMB2	RIOK1
SF3B5	TMEM147	TIMM17A
TMEM147	TSPO	WDR43

Only one module, *Lightcyan,* was preserved and associated with FEV1% in both lung tissue datasets but not in blood or sputum (Fig. 2, Table 2). Interestingly this module contained a set of genes related to B-lymphocyte biology (Additional file 4: Table S2).

### Consensus co-expression network of lung tissue and sputum

The network meta-analysis method does not assume module preservation, yet we identified multiple modules that are highly preserved in the other lung tissue dataset and the sputum datasets. To verify these results, we built a consensus network including the two lung tissue data sets and the sputum data set (as detailed in Additional file 1), which assumes preservation between datasets. Blood was not included, since none of the blood modules were related to the level of airflow limitation.

**Fig. 4:**
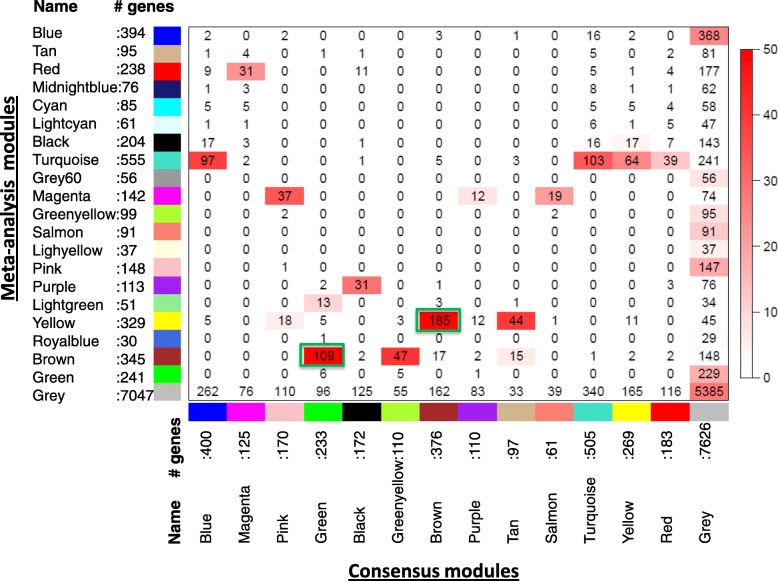
Comparison of the gene composition of the 14 consensus modules (columns) with the 21 meta-analysis modules (rows). Red color indicates higher concordance. When a meta-analysis module corresponded with more than one consensus module, the module with greater concordance is outlined with a green square. For further details on analytical method: (https://horvath.genetics.ucla.edu/html/CoexpressionNetwork/JMiller/)

The consensus WGCNA network identified 14 consensus modules (Additional file 6: Figure S2). Figure 4 shows the overlapping gene composition between these 14 consensus modules and the 21 WGCNA modules derived from lung tissue. The yellow WGCNA module and the brown consensus module shared 56.2% of their genes. The brown WGCNA module and the green consensus module shared 31.6% of genes. The WGCNA magenta module and the pink consensus module shared 26.1% of genes. In keeping with WGCNA results, the brown, green, and pink consensus modules were also associated with the severity of airflow limitation (Additional file 7: Figure S3).

### Core of co-expressed genes and related ontologies

**Fig. 5:**
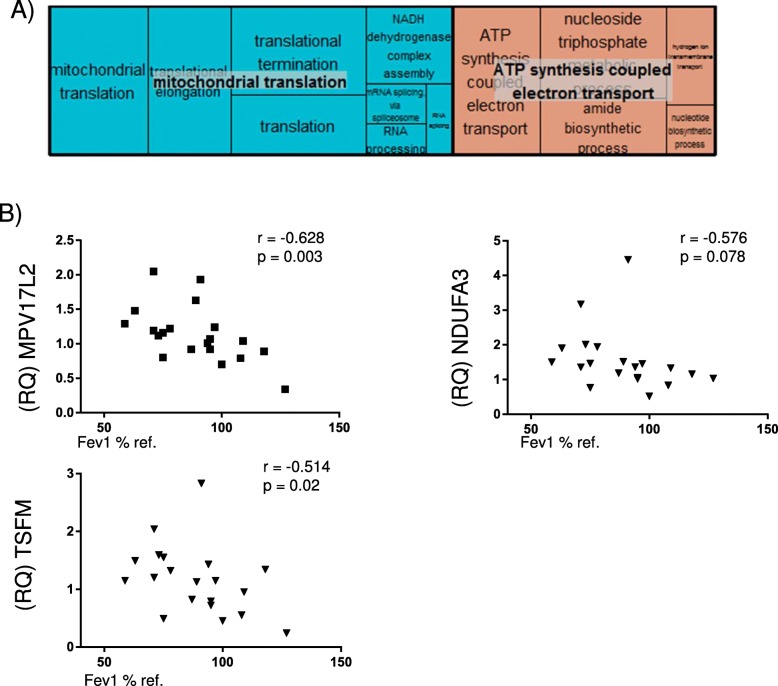
Analysis of 60 genes with concordance between the yellow meta-analysis module and brown consensus module and association with FEV_1._ (**a**) Visual summary (Treemap in REVIGO) of the gene ontologies. (**b**) Correlation (Spearman) between the qPCR expression level (RQ) of MPV17L2, NDUFA3 and TSFM and FEV_1_% predicted in 20 additional lung tissue samples

We found that, out of the 185 genes shared by the yellow WGCNA module and the brown consensus module, 60 genes were nominally associated with FEV1% predicted at *p* < 0.1 in lung tissue and sputum (Additional file 8: Table S4); Gene Ontology analysis showed enrichment in mitochondrial-related ontologies (Fig. 5a, Additional file 9: Table S5). Three mitochondrial-related genes were selected for qPCR validation on the basis of significant association with FEV_1_% in L1 and L2: NADH dehydrogenase 1 alpha subcomplex subunit 3 (NDUFA3), Ts Translation Elongation Factor, Mitochondrial (TSFM), and MPV17 Mitochondrial Inner Membrane Protein Like 2 (MPV17L2). Additional file 10: Figure S4 shows the gene expression of these three genes in L1 and L2 samples, and Fig. 5 shows that the relative expression of these genes (RQ) was also negatively correlated with the severity of airflow limitation in lung tissue obtained from 12 additional COPD patients and 8 controls.

## Discussion

We performed a gene co-expression network analysis in COPD datasets from two separate lung tissue studies, sputum, and blood. The main results show *(1)* robust gene module preservation between the two lung tissue datasets, with less preservation in sputum and even less in blood; *(2)* an association of the modules identified in the two lung tissue datasets and sputum (but not in blood) with the severity of airflow limitation (FEV1); and *(3)* that these findings were largely reproduced in a consensus WGCNA network. Taken together, these observations indicate that major changes in lung transcriptomics in patients with COPD are poorly mirrored in sputum and are unrelated to those determined in blood.

## Previous studies

Obeidat et al. recently used WGCNA to investigate blood transcriptomics in COPD patients (*n* = 238) [23]; however, our study used WGCNA to compare lung tissue, sputum and blood samples in COPD patients. While we found no gene modules associated with lung function in blood, Obeidat et al. identified 3 blood modules associated with FEV1. Differences between the two studies may be related to their larger sample size (238 vs. 121 patients). The gene ontology enrichments of these three blood modules [23] were different from those identified in preserved in lung and sputum in the present analysis, supporting that lung and blood are independent compartments in COPD [24], arguing against the so-called “spill-over” hypothesis, which states that the systemic manifestations of the disease are the result of the release to the circulation of lung inflammatory mediators [25].

## Interpretation of findings

Three modules associated with FEV1 were preserved in lung and sputum. These modules contained genes related to mitochondrial function, metabolic alterations, regulation of T cell activation, endocytosis and non-coding RNA metabolism. Some of these processes have been previously associated with COPD. For example, metabolic alterations are well documented in airway smooth muscle (ASM) cells [26], and CTS3 (a driver gene of the brown module) was previously reported to be causally associated with COPD [27]. Likewise, the loss of mitochondrial biogenesis (production of new mitochondria) and mitochondrial DNA (mtDNA) appear associated with a significantly lower body mass index and muscle mass in COPD [28, 29]. Similarly, inherited mtDNA haplotypes may also pre-dispose or confer susceptibility to COPD [30]. Little is known about the effects of mitochondrial translation or mRNA splicing in COPD, [31] although the role of mitochondrial and iron abnormalities has been described in relationship to a COPD genome-wide association gene *IREB2* [32]. We validated three of the key mitochondria-relevant genes encoded by the nuclear genome by qPCR. TSFM regulates the translation of the 13-mtDNA encoded genes in the mitochondrial matrix, whereas NDUFA3 encodes an accessory subunit of the mitochondrial membrane respiratory chain NADH dehydrogenase (Complex I). MPV17L2 is a mitochondrial inner membrane protein that regulates ribosomal assembly and protein synthesis in mitochondria [33].

Functionally, nearly every cell in the lung depends on mitochondrial metabolic activities, requiring a constant supply of energy from oxidative phosphorylation. Mitochondria are at the hub of cellular metabolism, regulating the continuous aerobic oxidation of fatty acids and consuming the end products of glucose, glutamine and amino acid degradation in order to aerobically produce ATP from oxygen and H_2_O [34]. An alteration in any of the three genes identified in this study may alter bioenergetic processes, mitochondrial shape, movement and cellular interactions. From studies of families with mutations in mitochondrial genes, it is known that impaired mitochondrial translation and impaired Complex I activity results in deficient ATP production and cellular energy deficit [35]. Accordingly, in COPD mitochondrial abnormalities have been associated with excessive production of mitochondrial Reactive Oxygen Species (mROS) and abnormalities in ATP production that in turn lead to enhanced inflammation and cell hyperproliferation [36]. Further studies are required to know if the abnormal mROS observed are related to defects in translation or whether these defects are consequences of the continuous exposure to noxious gases and particles, such as tobacco smoke, in the lung.

Finally, we identified only one module in the meta-analysis which was preserved and associated with FEV_1_% predicted in the two lung tissue datasets (light-yellow), but not in any other compartment. This module contained B-cell related genes that have been previously associated with the presence of emphysema and/or the severity of airflow limitation [3, 16, 37]. Therefore, our findings here suggest that the B-cell component of COPD cannot be readily identified in sputum or blood.

## Strengths and limitations

The fact that the two lung studies were performed in different countries using different array platforms but still showed good preservation of co-expression and association with airflow limitation is a strength of our study. This reflects both the reproducibility of the transcriptomic changes associated with COPD once the potential confounding effect of active smoking is removed and that network-based transcriptomic meta-analysis is a suitable tool to cope with methodological differences [14, 38]. Among the limitations of our study, we acknowledge that only blood and sputum, but not lung tissue, data were obtained simultaneously from the same patients. This is the reason why in the current analysis we used WGCNA, as previous works have described the ability of the method to assess module preservation across different tissues and even across different species, overcoming the limitation of using different subjects [15, 39]. The fact that we do not observe preservation of modules across sputum and blood that are from the same individuals also supports the conclusion that there are co-expressed genes associated with the severity of airflow limitation only in lung and sputum.Because our study was observational, functional evidence based on animal models or longitudinal human studies are required to validate the clinical relevance of our observations. We acknowledge that it would have been desirable to analyse the mitochondrial gene expression in relation to diverse COPD clinical parameters (i.e. exacerbations, treatment, prognosis or blood exam measures) but this data was not available for the current study and should be addressed in future investigations.

Finally, in this study it is unclear how much of the difference in gene expression between samples was due to differences in cellular composition vs. differences in expression across cell types.

## Conclusions

Using gene expression correlation-based network analysis, we identified modules of co-expressed genes that were preserved and associated with the severity of airflow limitation in lung tissue and sputum, but not in blood samples, suggesting that the systemic component in COPD is independently regulated. The fact that one of the preserved modules associated with FEV1 was enriched in mitochondria-related genes supports a role for mitochondrial dysfunction in the pathobiology of COPD.

## Additional files

Additional file 1: On-line supplement methods. (DOCX 37 kb)

Additional file 2: **Figure S1.** Association between blood gene modules and lung function. The module definition was performed based on the blood dataset. (DOCX 41 kb)

Additional file 3: **Table S1.** Genes in Brown, Yellow, Magenta and LightCyan modules. (PDF 51 kb)

Additional file 4: **Table S2.** Gene Ontology enrichment in Yellow, Brown, and Magenta modules. (PDF 52 kb)

Additional file 5: **Table S3.** KEGG pathway enrichment in Yellow, Brown, and Magenta modules. (PDF 36 kb)

Additional file 6: **Figure S2.** Dendrogram of consensus co-expression network including Lung Tissue-1, Lung Tissue-2 and Sputum datasets. (DOCX 48 kb)

Additional file 7: **Figure S3.** Association between consensus gene modules and lung function in each cohort. (DOCX 96 kb)

Additional file 8: **Table S4.** Core 60 genes in common between the yellow meta-analysis module and brown consensus module with the *p*-value <0.1 for association with FEV1 % predicted. (PDF 38 kb)

Additional file 9: **Table S5.** Gene Ontology enrichment for the core 60 genes. (PDF 33 kb)

Additional file 10: **Figure S4.** Correlation of the gene expression of MPV17L2, NDUFA3 and TSFM with FEV1 % predicted in L1 (A) and L2 (B). (DOCX 98 kb)

## Abbreviations

B: Blood dataset
BMI: Body mass index
COPD: Chronic obstructive pulmonary disease
ECLIPSE: Evaluation of copd longitudinally to identify predictive surrogate end-points
L: Lung tissue dataset
S: Induced sputum dataset
WGCNA: Weighted gene co-expression network analysis
