# Supplementary material for: Do sputum or circulating blood samples reflect the pulmonary transcriptomic differences of COPD patients? A multi-tissue transcriptomic network META-analysis
Source: Respir Res. 2019 Jan 8;20:5. doi: 10.1186/s12931-018-0965-y (PMC6325784; doi:10.1186/s12931-018-0965-y)
Supplement: Supplementary file 1 — On-line supplement methods. (DOCX 37 kb) [file 12931_2018_965_MOESM1_ESM.docx]

**DO SPUTUM OR CIRCULATING BLOOD SAMPLES REFLECT THE PULMONARY TRANSCRIPTOMIC CHANGES OF COPD PATIENTS?**

**a multi-tissue transcriptomic network meta-analysis**

**Authors:** Rosa Faner^1^, Jarrett D. Morrow^2^, Sandra Casas-Recasens^1^, Suzanne M. Cloonan^3^, Guillaume Noell^1^, Alejandra López-Giraldo^1,4^, Ruth Tal-Singer^5^, Bruce Miller^5^, Edwin K. Silverman^2^, Alvar Agustí^1,4^, Craig P. Hersh^2^.

^1^ Centro de Investigación Biomédica en Red de Enfermedades Respiratorias (CIBERES), Madrid, Spain.

^2^ Channing Division of Network Medicine. Brigham and Women's Hospital. Boston, US.

^3^ Division of Pulmonary and Critical Care Medicine, Joan and Sanford I. Weill Department of Medicine, Weill Cornell Medical College, New York, NY, USA.

^4^ Institute Clinic Respiratory, Pulmonary Service, Hospital Clinic, IDIBAPS, University of Barcelona, Spain.

^5^ GSK R&D, King of Prussia, PA, USA.

**ON-LINE SUPPLEMENT METHODS**

**Study subjects**

This meta-analysis used four transcriptomic datasets generated in three different studies.

Lung tissue from the Faner et al. study [1] (L1) was obtained from COPD patients, diagnosed using the GOLD criteria [2], who underwent thoracic surgery for cancer resection or lung transplantation. Lung tissue from Morrow et al. [3] (L2) study was collected from subjects with severe COPD and controls with normal spirometry undergoing thoracic surgery for lung transplantation, lung volume reduction, or nodule resection at three medical centers.

ECLIPSE was a 3-year multi-center longitudinal study to identify novel endpoints in COPD; the methodology has been previously described [4]. Induced sputum (S) and blood samples (B) were obtained in a subset of 148 ex-smokers with COPD at 14 sites at the start of the study [5, 6]. Selecting Caucasian individuals with samples of sufficient quality for expression microarray analysis lead to the 121 subjects included in the present analysis. To avoid the confounding effect of active smoking on lung transcriptomics [7], all studied individuals in the 4 data sets were former smokers, abstinent at least one month before tissue sampling.

qPCR validation subjects consisted in n=12 COPD former smokers: Age (67.8±8.2), pack/year (52.4±23.6), FEV1/FVC (61.7±5.9), FEV1 % ref. (82.8±17.9), FVC %ref. (98.8±18.8) and n=8 Controls: Age (66.0±12.3), pack/year (37.5±37.5), FEV1/FVC (76.4±4.7), FEV1 % ref. (100.6±10.1), FVC %ref. (93.7±12.0).

**Weighted gene co-expression network meta-analysis**

Weighted gene co-expression networks were built and meta-analyzed using the WGCNA R package

**Consensus network**

Linear regression with adjustment for gender and BMI was used to identify genes associated with FEV_1_ % predicted in the three datasets [11]. In the sputum dataset, we additionally performed the linear regressions for association the percentage of macrophages or neutrophils, adjusted with BMI and FEV_1_ % predicted.

**Supplementary table 1:** Genes in Brown, Yellow, Magenta and LightCyan modules.

**Supplementary table 2:** Gene Ontology enrichment in Yellow, Brown, and Magenta modules.

**Supplementary table 3:** KEGG pathway enrichment in Yellow, Brown, and Magenta modules.

**Supplementary table 4:** Core 60 genes in common between the yellow meta-analysis module and brown consensus module with the p-value <0.1 for association with FEV1 % predicted. It is included the official gene symbol (Gene.Symbol), the number of gene ID (gene_id), the gene significance (GS) values with the values of FEV1 % predicted in each data-set (i.e. GS.set1.FEV1.PP is for LT-1, GS.set2.FEV1.PP is for LT-1 is for LT-2 and GS.set3.FEV1.PP is for Sputum-Eclipse), and the p values of the gene significant calculation in each data-set (i.e. p.GS.set1.FEV1.PP is the p value of the gene significance for LT-1).

**Supplementary table 5:** Gene Ontology enrichment for the core 60 genes.

**SUPPLEMENTARY FIGURE LEGENDS**

**Supplementary Figure 1**: Association between blood gene modules and lung function. The module definition was performed based on the blood dataset. Heat-map shows the p-values (and effect estimates) of the linear regression of each module eigengene with FEV1 % predicted in each of the four datasets. Yellow denotes lower p-values and blue higher p-values.

**Supplementary Figure 2**: Dendrogram of consensus co-expression network including Lung Tissue-1, Lung Tissue-2 and Sputum datasets. The bottom shows the 14 consensus modules in different colors.

**Supplementary Figure 3:** Association between consensus gene modules and lung function in each cohort. Heat-map shows the p-values (and effect estimates) of the linear regression of each module eigengene with FEV1 % predicted in each of the four datasets. Yellow denotes lower p-values and blue higher p-values.

**Supplementary Figure 4:** Correlation of the gene expression of MPV17L2, NDUFA3 and TSFM with FEV1 % predicted in L1 (A) and L2 (B). Blue dots denote samples from control individuals, black dots COPD cases.

**References**

1. Faner R, Cruz T, Casserras T, Lopez-Giraldo A, Noell G, Coca I, Tal-Singer R, Miller B, Rodriguez-Roisin R, Spira A, et al: **Network Analysis of Lung Transcriptomics Reveals a Distinct B Cell Signature in Emphysema.** *American journal of respiratory and critical care medicine* 2016.

2. Vestbo J, Hurd SS, Agusti AG, Jones PW, Vogelmeier C, Anzueto A, Barnes PJ, Fabbri LM, Martinez FJ, Nishimura M, et al: **Global strategy for the diagnosis, management, and prevention of chronic obstructive pulmonary disease: GOLD executive summary.** *American journal of respiratory and critical care medicine* 2013, **187:**347-365.

3. Morrow JD, Zhou X, Lao T, Jiang Z, DeMeo DL, Cho MH, Qiu W, Cloonan S, Pinto-Plata V, Celli B, et al: **Functional interactors of three genome-wide association study genes are differentially expressed in severe chronic obstructive pulmonary disease lung tissue.** *Sci Rep* 2017, **7:**44232.

4. Vestbo J, Anderson W, Coxson HO, Crim C, Dawber F, Edwards L, Hagan G, Knobil K, Lomas DA, MacNee W, et al: **Evaluation of COPD Longitudinally to Identify Predictive Surrogate End-points (ECLIPSE).** *The European respiratory journal* 2008, **31:**869-873.

5. Singh D, Fox SM, Tal-Singer R, Plumb J, Bates S, Broad P, Riley JH, Celli B: **Induced sputum genes associated with spirometric and radiological disease severity in COPD ex-smokers.** *Thorax* 2011, **66:**489-495.

6. Singh D, Fox SM, Tal-Singer R, Bates S, Riley JH, Celli B: **Altered gene expression in blood and sputum in COPD frequent exacerbators in the ECLIPSE cohort.** *PLoS One* 2014, **9:**e107381.

7. Bosse Y, Postma DS, Sin DD, Lamontagne M, Couture C, Gaudreault N, Joubert P, Wong V, Elliott M, van den Berge M, et al: **Molecular signature of smoking in human lung tissues.** *Cancer Res* 2012, **72:**3753-3763.

8. Langfelder P, Horvath S: **WGCNA: an R package for weighted correlation network analysis.** *BMC bioinformatics* 2008, **9:**559.

9. Malki K, Tosto MG, Jumabhoy I, Lourdusamy A, Sluyter F, Craig I, Uher R, McGuffin P, Schalkwyk LC: **Integrative mouse and human mRNA studies using WGCNA nominates novel candidate genes involved in the pathogenesis of major depressive disorder.** *Pharmacogenomics* 2013, **14:**1979-1990.

10. Langfelder P, Luo R, Oldham MC, Horvath S: **Is my network module preserved and reproducible?** *PLoS Comput Biol* 2011, **7:**e1001057.

11. Ritchie ME, Phipson B, Wu D, Hu Y, Law CW, Shi W, Smyth GK: **limma powers differential expression analyses for RNA-sequencing and microarray studies.** *Nucleic Acids Res* 2015, **43:**e47.

12. Miller JA, Horvath S, Geschwind DH: **Divergence of human and mouse brain transcriptome highlights Alzheimer disease pathways.** *Proc Natl Acad Sci U S A* 2010, **107:**12698-12703.
