# Supplementary figures and images for: Do sputum or circulating blood samples reflect the pulmonary transcriptomic differences of COPD patients? A multi-tissue transcriptomic network META-analysis
Source: Respir Res. 2019 Jan 8;20:5. doi: 10.1186/s12931-018-0965-y (PMC6325784; doi:10.1186/s12931-018-0965-y)

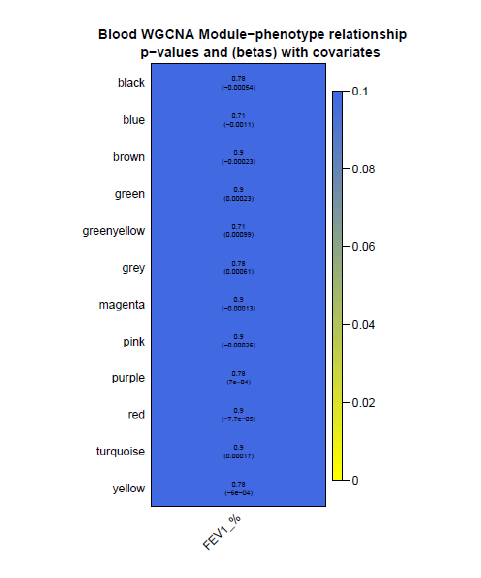

Supplement: Supplementary file 2 — Figure S1. Association between blood gene modules and lung function. The module definition was performed based on the blood dataset. (DOCX 41 kb) [file 12931_2018_965_MOESM2_ESM.docx]

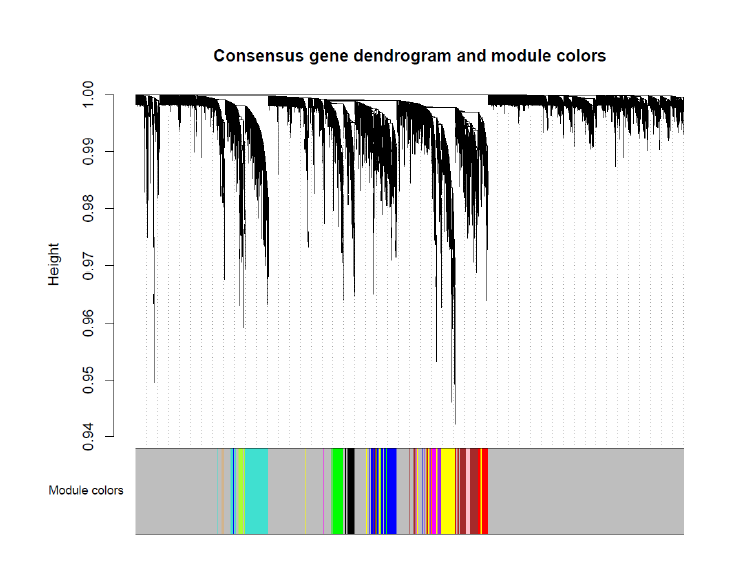

Supplement: Supplementary file 6 — Figure S2. Dendrogram of consensus co-expression network including Lung Tissue-1, Lung Tissue-2 and Sputum datasets. (DOCX 48 kb) [file 12931_2018_965_MOESM6_ESM.docx]

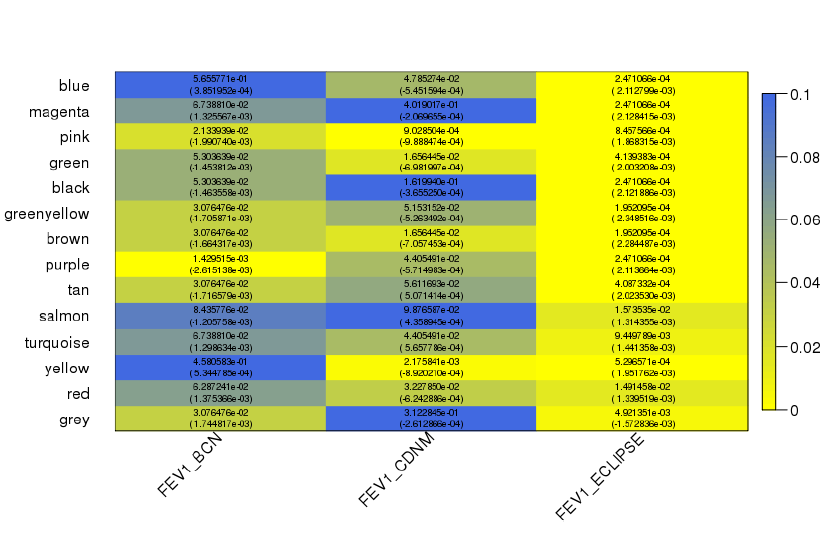

Supplement: Supplementary file 7 — Figure S3. Association between consensus gene modules and lung function in each cohort. (DOCX 96 kb) [file 12931_2018_965_MOESM7_ESM.docx]

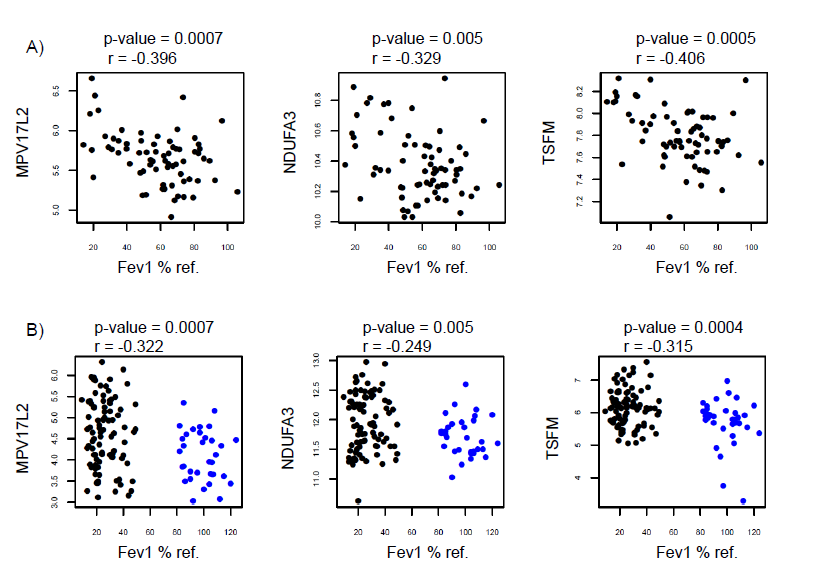

Supplement: Supplementary file 10 — Figure S4. Correlation of the gene expression of MPV17L2, NDUFA3 and TSFM with FEV1 % predicted in L1 (A) and L2 (B). (DOCX 98 kb) [file 12931_2018_965_MOESM10_ESM.docx]
