# Supplementary material for: Do sputum or circulating blood samples reflect the pulmonary transcriptomic differences of COPD patients? A multi-tissue transcriptomic network META-analysis
Source: Respir Res. 2019 Jan 8;20:5. doi: 10.1186/s12931-018-0965-y (PMC6325784; doi:10.1186/s12931-018-0965-y)
Supplement: Supplementary file 3 — Table S1. Genes in Brown, Yellow, Magenta and LightCyan modules. (PDF 51 kb) [file 12931_2018_965_MOESM3_ESM.pdf]

Table S1

Genes in Brown module	
**Entrez.Gene**	**Gene.symbol**
35	ACADS
11332	ACOT7
53	ACP2
54	ACP5
55803	ADAP2
140	ADORA3
83440	ADPGK
175	AGA
56894	AGPAT3
199	AIF1
231	AKR1B1
226	ALDOA
162	AP1B1
1174	AP1S1
1175	AP2S1
327	APEH
27301	APEX2
27350	APOBEC3C
341	APOC1
348	APOE
58504	ARHGAP22
257106	ARHGAP30
397	ARHGDIB
411	ARSB
430	ASCL2
57168	ASPHD2
84938	ATG4C
10533	ATG7
475	ATOX1
523	ATP6V1A
528	ATP6V1C1
9550	ATP6V1G1
10134	BCAP31
586	BCAT1
641	BLM
29760	BLNK
9577	BRE
84446	BRSK1
712	C1QA
713	C1QB
714	C1QC
719	C3AR1
822	CAPG
114769	CARD16
64170	CARD9
838	CASP5
55704	CCDC88A

Genes in Yellow Module	
**Entrez.Gene**	**Gene.symbol**
57001	ACN9
51094	ADIPOR1
57085	AGTRAP
191	AHCY
29880	ALG5
84266	ALKBH7
51529	ANAPC11
353	APRT
439	ASNA1
471	ATIC
516	ATP5G1
517	ATP5G2
518	ATP5G3
521	ATP5I
9551	ATP5J2
10632	ATP5L
8992	ATP6V0E1
51382	ATP6V1D
9296	ATP6V1F
64756	ATPAF1
54998	AURKAIP1
57099	AVEN
8815	BANF1
645	BLVRB
388962	BOLA3
708	C1QBP
27101	CACYBP
133957	CCDC127
891	CCNB1
899	CCNF
10694	CCT8
246184	CDC26
55038	CDCA4
1019	CDK4
1020	CDK5
118487	CHCHD1
51142	CHCHD2
51550	CINP
55847	CISD1
493856	CISD2
284106	CISD3
1164	CKS2
1207	CLNS1A
8192	CLPP
10175	CNIH
29097	CNIH4
10330	CNPY2

Genes in Magenta Module	
**Entrez.Gene**	**Gene.symbol**
6059	ABCE1
10061	ABCF2
2182	ACSL4
262	AMD1
81873	ARPC5L
79572	ATP13A3
10538	BATF
56647	BCCIP
9790	BMS1
55299	BRIX1
705	BYSL
9689	BZW1
28969	BZW2
868	CBLB
79080	CCDC86
10576	CCT2
908	CCT6A
23580	CDC42EP4
10153	CEBPZ
79145	CHCHD7
84916	CIRH1A
51202	DDX47
27000	DNAJC2
1876	E2F6
9521	EEF1E1
30844	EHD4
1965	EIF2S1
8669	EIF3J
1981	EIF4G1
56943	ENY2
2120	ETV6
79850	FAM57A
2553	GABPB1
2617	GARS
2773	GNAI3
29889	GNL2
64841	GNPNAT1
54865	GPATCH4
53831	GPR84
80273	GRPEL1
83743	GRWD1
23560	GTPBP4
3014	H2AFX
57817	HAMP
3251	HPRT1
3419	IDH3A
3428	IFI16

6368	CCL23
9332	CD163
131450	CD200R1
146722	CD300LF
945	CD33
1043	CD52
963	CD53
971	CD72
972	CD74
8832	CD84
942	CD86
83461	CDCA3
83879	CDCA7
400916	CHCHD10
55743	CHFR
66005	CHID1
4261	CIITA
64581	CLEC7A
54982	CLN6
81570	CLPB
54918	CMTM6
112616	CMTM7
55748	CNDP2
10695	CNPY3
51397	COMMD10
29099	COMMD9
27235	COQ2
57175	CORO1B
23406	COTL1
54504	CPVL
8804	CREG1
56253	CRTAM
1436	CSF1R
1438	CSF2RA
1476	CSTB
5476	CTSA
1508	CTSB
1075	CTSC
1509	CTSD
1520	CTSS
51167	CYB5R4
1535	CYBA
1536	CYBB
1593	CYP27A1
27071	DAPP1
55157	DARS2
28988	DBNL
1633	DCK
28960	DCPS
54165	DCUN1D1

150684	COMMD1
23412	COMMD3
28991	COMMD5
170622	COMMD6
54951	COMMD8
57017	COQ9
9377	COX5A
1329	COX5B
1349	COX7B
9419	CRIPT
10675	CSPG5
7818	DAP3
1622	DBI
79077	DCTPP1
1652	DDT
51009	DERL2
29103	DNAJC15
23234	DNAJC9
1798	DPAGT1
51611	DPH5
8818	DPM2
54344	DPM3
92675	DTD1
54935	DUSP23
8798	DYRK4
10969	EBNA1BP2
1892	ECHS1
55520	ELAC1
84337	ELOF1
10436	EMG1
51614	ERGIC3
9156	EXO1
51013	EXOSC1
23016	EXOSC7
11124	FAF1
2184	FAH
84908	FAM136A
10667	FARS2
9158	FIBP
80308	FLAD1
139341	FUNDC1
65991	FUNDC2
2395	FXN
90480	GADD45GIP1
23464	GCAT
79833	GEMIN6
2671	GFER
2733	GLE1
51053	GMNN
8833	GMPS

3460	IFNGR2
3554	IL1R1
3556	IL1RAP
8808	IL1RL2
10527	IPO7
9933	KIAA0020
353514	LILRA5
9516	LITAF
116844	LRG1
55379	LRRC59
10459	MAD2L2
84549	MAK16
4141	MARS
92935	MARS2
4234	METTL1
4247	MGAT2
84365	MKI67IP
64983	MRPL32
51154	MRTO4
10797	MTHFD2
51388	NIP7
64318	NOC3L
79954	NOL10
64434	NOM1
51491	NOP16
4839	NOP2
8204	NRIP1
54888	NSUN2
5036	PA2G4
55003	PAK1IP1
114299	PALM2
201626	PDE12
9124	PDLIM1
118987	PDZD8
23481	PES1
51105	PHF20L1
23761	PISD
57480	PLEKHG1
5352	PLOD2
56902	PNO1
87178	PNPT1
9533	POLR1C
5433	POLR2D
10940	POP1
10105	PPIF
9128	PRPF4
5771	PTPN2
51651	PTRH2
5806	PTX3
54517	PUS7

80821	DDHD1
65992	DDRGK1
79961	DENND2D
207063	DHRSX
23312	DMXL2
85479	DNAJC5B
1777	DNASE2
1794	DOCK2
9046	DOK2
29952	DPP7
84062	DTNBP1
79180	EFHD2
1978	EIF4EBP1
84034	EMILIN2
23071	ERP44
2123	EVI2A
2170	FABP3
81889	FAHD1
65990	FAM173A
84331	FAM195A
253725	FAM21C
51571	FAM49B
84191	FAM96A
55711	FAR2
79675	FASTKD1
26263	FBXO22
2213	FCGR2B
2217	FCGRT
2235	FECH
83706	FERMT3
221472	FGD2
23307	FKBP15
2359	FPR3
2517	FUCA1
2530	FUT8
53827	FXYD5
26301	GBGT1
26157	GIMAP2
2720	GLB1
2745	GLRX
51022	GLRX2
2760	GM2A
59345	GNB4
10007	GNPDA1
2799	GNS
2821	GPI
10457	GPNMB
7107	GPR137B
2857	GPR34
8477	GPR65

404672	GTF2H5
2971	GTF3A
10456	HAX1
374659	HDDC3
23593	HEBP2
192286	HIGD2A
3094	HINT1
84681	HINT2
3145	HMBS
3281	HSBP1
285148	IAH1
3416	IDE
3704	ITPA
84522	JAGN1
200185	KRTCAP2
3945	LDHB
27257	LSM1
84967	LSM10
27258	LSM3
25804	LSM4
23658	LSM5
51690	LSM7
84316	LSMD1
57128	LYRM4
55110	MAGOHB
28985	MCTS1
4201	MEA1
51003	MED31
55897	MESP1
29081	METTL5
51108	METTL9
4258	MGST2
4282	MIF
79682	MLF1IP
84769	MPV17L2
78988	MRP63
65003	MRPL11
6182	MRPL12
28998	MRPL13
29088	MRPL15
63875	MRPL17
219927	MRPL21
29093	MRPL22
6150	MRPL23
51264	MRPL27
11222	MRPL3
64981	MRPL34
51318	MRPL35
64979	MRPL36
51253	MRPL37

5822	PWP2
5901	RAN
55131	RBM28
83732	RIOK1
84154	RPF2
6165	RPL35A
23212	RRS1
22904	SBNO2
81929	SEH1L
10509	SEMA4B
12	SERPINA3
130367	SGPP2
6510	SLC1A5
23516	SLC39A14
84102	SLC41A2
9497	SLC4A7
6632	SNRPD1
80176	SPSB1
153443	SRFBP1
140809	SRXN1
6774	STAT3
10923	SUB1
6875	TAF4B
6897	TARS
6996	TDG
54881	TEX10
10440	TIMM17A
7076	TIMP1
8797	TNFRSF10A
27242	TNFRSF21
8740	TNFSF14
79155	TNIP2
10953	TOMM34
55720	TSR1
221830	TWISTNB
7334	UBE2N
9816	URB2
9100	USP10
51118	UTP11L
9218	VAPA
23160	WDR43
55854	ZC3H15
51114	ZDHHC9
7756	ZNF207
51042	ZNF593

63940	GPSM3
2876	GPX1
57476	GRAMD1B
2885	GRB2
2896	GRN
2937	GSS
2954	GSTZ1
2990	GUSB
9555	H2AFY
84868	HAVCR2
10870	HCST
3073	HEXA
3074	HEXB
3108	HLA-DMA
3113	HLA-DPA1
3115	HLA-DPB1
51155	HN1
3176	HNMT
84343	HPS3
10553	HTATIP2
203100	HTRA4
84329	HVCN1
3417	IDH1
3418	IDH2
83982	IFI27L2
10643	IGF2BP3
3606	IL18
10788	IQGAP2
3394	IRF8
3689	ITGB2
8514	KCNAB2
3762	KCNJ5
3778	KCNMA1
57650	KIAA1524
89857	KLHL6
8564	KMO
8942	KYNU
3903	LAIR1
254251	LCORL
3965	LGALS9
5641	LGMN
10859	LILRB1
11006	LILRB4
3988	LIPA
10960	LMAN2
84695	LOXL3
9404	LPXN
123355	LRRC28
9450	LY86
4074	M6PR

64976	MRPL40
64975	MRPL41
84311	MRPL45
51642	MRPL48
51258	MRPL51
122704	MRPL52
64963	MRPS11
6183	MRPS12
63931	MRPS14
64960	MRPS15
51021	MRPS16
51023	MRPS18C
54460	MRPS21
51649	MRPS23
64432	MRPS25
51650	MRPS33
65993	MRPS34
51081	MRPS7
22921	MSRB2
23788	MTCH2
10651	MTX2
51079	NDUFA13
4695	NDUFA2
4696	NDUFA3
4700	NDUFA6
4701	NDUFA7
4702	NDUFA8
4704	NDUFA9
4706	NDUFAB1
91942	NDUFAF2
4707	NDUFB1
54539	NDUFB11
4708	NDUFB2
4713	NDUFB7
4722	NDUFS3
4725	NDUFS5
374291	NDUFS7
4731	NDUFV3
55651	NHP2
4898	NRD1
50814	NSDHL
25936	NSL1
197370	NSMCE1
134492	NUDCD2
4521	NUDT1
84304	NUDT22
51686	OAZ3
29789	OLA1
29095	ORMDL2
55611	OTUB1

4125	MAN2B1
79109	MAPKAP1
8685	MARCO
57192	MCOLN1
4190	MDH1
4199	ME1
4200	ME2
79828	METTL8
64747	MFSD1
64780	MICAL1
4277	MICB
6945	MLX
23531	MMD
10198	MPHOSPH9
4354	MPP1
4358	MPV17
51338	MS4A4A
64231	MS4A6A
58475	MS4A7
4522	MTHFD1
4644	MYO5A
4668	NAGA
55577	NAGK
4669	NAGLU
51172	NAGPA
4690	NCK1
55707	NECAP2
10783	NEK6
9603	NFE2L3
387921	NHLRC3
58484	NLRC4
80896	NPL
10062	NR1H3
4905	NSF
8439	NSMAF
26578	OSTF1
5025	P2RX4
5027	P2RX7
64098	PARVG
5106	PCK2
5165	PDK3
8566	PDXK
5226	PGD
118788	PIK3AP1
29992	PILRA
7941	PLA2G7
5330	PLCB2
55163	PNPO
57804	POLD4
55012	PPP2R3C

5034	P4HB
5092	PCBD1
5096	PCCB
5111	PCNA
9141	PDCD5
5147	PDE6D
51248	PDZD11
5191	PEX7
5203	PFDN4
25796	PGLS
5245	PHB
29085	PHPT1
5303	PIN4
8228	PNPLA4
5434	POLR2E
5435	POLR2F
51728	POLR3K
51367	POP5
10248	POP7
27068	PPA2
5479	PPIB
10450	PPIE
5518	PPP2R1A
5683	PSMA2
5685	PSMA4
5686	PSMA5
5688	PSMA7
5690	PSMB2
5691	PSMB3
5693	PSMB5
10213	PSMD14
5715	PSMD9
51495	PTPLAD1
5889	RAD51C
5902	RANBP1
9978	RBX1
25996	REXO2
10535	RNASEH2A
79621	RNASEH2B
84153	RNASEH2C
9616	RNF7
140823	ROMO1
51121	RPL26L1
116832	RPL39L
91582	RPS19BP1
51065	RPS27L
6193	RPS5
83694	RPS6KL1
51389	RWDD1
10055	SAE1

5538	PPT1
5567	PRKACB
5724	PTAFR
5800	PTPRO
150962	PUS10
29108	PYCARD
23518	R3HDM1
8934	RAB7L1
5910	RAP1GDS1
5912	RAP2B
83937	RASSF4
5965	RECQL
5993	RFX5
6001	RGS10
391	RHOG
6039	RNASE6
8635	RNASET2
55819	RNF130
58528	RRAGD
950	SCARB2
51097	SCCPDH
59342	SCPEP1
113675	SDSL
23231	SEL1L3
83852	SETDB2
375035	SFT2D2
81537	SGPP1
79801	SHCBP1
6614	SIGLEC1
27036	SIGLEC7
27180	SIGLEC9
55423	SIRPG
56833	SLAMF8
51296	SLC15A3
63910	SLC17A9
6507	SLC1A3
8402	SLC25A11
91137	SLC25A46
55315	SLC29A3
56606	SLC2A9
1317	SLC31A1
206358	SLC36A1
145389	SLC38A6
153129	SLC38A9
201266	SLC39A11
283537	SLC46A3
6539	SLC6A12
23428	SLC7A8
6546	SLC8A1
11309	SLCO2B1

51282	SCAND1
6390	SDHB
10952	SEC61B
23480	SEC61G
27230	SERP1
83443	SF3B5
119559	SFXN4
6472	SHMT2
51629	SLC25A39
23583	SMUG1
64754	SMYD3
79622	SNRNP25
6633	SNRPD2
6635	SNRPE
6636	SNRPF
60559	SPCS3
8636	SSNA1
6747	SSR3
10534	SSSCA1
55240	STEAP3
30968	STOML2
201254	STRA13
8677	STX10
51657	STYXL1
6612	SUMO3
6834	SURF1
6881	TAF10
9519	TBPL1
90843	TCEAL8
6923	TCEB2
29087	THYN1
26519	TIMM10
26517	TIMM13
10245	TIMM17B
26521	TIMM8B
23423	TMED3
84233	TMEM126A
55863	TMEM126B
51524	TMEM138
85014	TMEM141
10430	TMEM147
153339	TMEM167A
80775	TMEM177
374882	TMEM205
29100	TMEM208
51259	TMEM216
124446	TMEM219
56993	TOMM22
9540	TP53I3
11257	TP53TG1

6641	SNTB1
6642	SNX1
29886	SNX8
6646	SOAT1
79644	SRD5A3
7903	ST8SIA4
3925	STMN1
56670	SUCNR1
64420	SUSD1
203328	SUSD3
6850	SYK
9144	SYNGR2
10460	TACC3
6892	TAPBP
128387	TATDN3
6916	TBXAS1
10312	TCIRG1
6948	TCN2
55775	TDP1
22797	TFEC
29844	TFPT
7037	TFRC
7045	TGFBI
7073	TIAL1
51284	TLR7
51311	TLR8
53346	TM6SF1
50999	TMED5
80194	TMEM134
55281	TMEM140
55248	TMEM206
144110	TMEM86A
27010	TPK1
7172	TPMT
1200	TPP1
8717	TRADD
9830	TRIM14
7226	TRPM2
51393	TRPV2
283237	TTC9C
8458	TTF2
1890	TYMP
140739	UBE2F
7444	VRK2
143187	VTI1A
57705	WDFY4
84272	YIPF4
84885	ZDHHC12

7167	TPI1
51002	TPRKB
51693	TRAPPC2L
126003	TRAPPC5
51499	TRIAP1
54952	TRNAU1AP
10102	TSFM
26262	TSPAN17
7264	TSTA3
84817	TXNDC17
127253	TYW3
10422	UBAC1
7993	UBXN8
7381	UQCRB
7384	UQCRC1
27089	UQCRQ
7389	UROD
7390	UROS
51096	UTP18
8409	UXT
8673	VAMP8
7419	VDAC3
79001	VKORC1
84313	VPS25
55255	WDR41
7511	XPNPEP1
57510	XPO5
10897	YIF1A
339487	ZBTB8OS
51304	ZDHHC3
55146	ZDHHC4
9326	ZNHIT3
