# Supplementary material for: Do sputum or circulating blood samples reflect the pulmonary transcriptomic differences of COPD patients? A multi-tissue transcriptomic network META-analysis
Source: Respir Res. 2019 Jan 8;20:5. doi: 10.1186/s12931-018-0965-y (PMC6325784; doi:10.1186/s12931-018-0965-y)
Supplement: Supplementary file 4 — Table S2. Gene Ontology enrichment in Yellow, Brown, and Magenta modules. (PDF 52 kb) [file 12931_2018_965_MOESM4_ESM.pdf]

Table S2

Gene Ontology Description and: ID	# genes	p value	fdr p value
**Brown module**			
lysosome organization::GO:0007040	9	1.41E-07	9.85E-05
lytic vacuole organization::GO:0080171	9	1.41E-07	9.85E-05
endocytosis::GO:0006897	31	8.52E-08	9.85E-05
oxidoreduction coenzyme metabolic process::GO:0006733	13	6.00E-07	0.0003144
pyridine nucleotide metabolic process::GO:0019362	12	1.18E-06	0.0004128
nicotinamide nucleotide metabolic process::GO:0046496	12	1.18E-06	0.0004128
aminoglycan catabolic process::GO:0006026	9	1.96E-06	0.0004406
positive regulation of T cell activation::GO:0050870	15	1.53E-06	0.0004406
positive regulation of homotypic cell-cell adhesion::GO:0034112	15	1.97E-06	0.0004406
positive regulation of leukocyte cell-cell adhesion::GO:1903039	15	2.10E-06	0.0004406
carboxylic acid metabolic process::GO:0019752	38	2.45E-06	0.000466
transition metal ion transport::GO:0000041	11	3.02E-06	0.0005281
pattern recognition receptor signaling pathway::GO:0002221	14	4.13E-06	0.0006663
glycosphingolipid metabolic process::GO:0006687	9	4.63E-06	0.0006728
antigen processing and presentation of exogenous peptide antigen via MHC class II::GO:0019886	10	4.82E-06	0.0006728
glycosaminoglycan catabolic process::GO:0006027	8	9.91E-06	0.001222
T cell proliferation::GO:0042098	13	9.82E-06	0.001222
cellular cation homeostasis::GO:0030003	24	1.18E-05	0.001372
cellular ion homeostasis::GO:0006873	24	1.67E-05	0.001801
cation homeostasis::GO:0055080	26	1.80E-05	0.001801
ion homeostasis::GO:0050801	28	1.79E-05	0.001801
coenzyme biosynthetic process::GO:0009108	10	2.95E-05	0.00247
glycolipid metabolic process::GO:0006664	10	2.95E-05	0.00247
inorganic ion homeostasis::GO:0098771	26	2.75E-05	0.00247
pyridine-containing compound biosynthetic process::GO:0072525	5	3.14E-05	0.002531
transferrin transport::GO:0033572	6	3.26E-05	0.002531
ferric iron transport::GO:0015682	6	4.49E-05	0.003244
trivalent inorganic cation transport::GO:0072512	6	4.49E-05	0.003244
dicarboxylic acid metabolic process::GO:0043648	9	5.14E-05	0.003593
toll-like receptor signaling pathway::GO:0002224	11	6.26E-05	0.004234
receptor-mediated endocytosis::GO:0006898	15	7.54E-05	0.004644
lymphocyte aggregation::GO:0071593	20	7.46E-05	0.004644
cellular metal ion homeostasis::GO:0006875	21	7.66E-05	0.004644
metal ion homeostasis::GO:0055065	23	8.31E-05	0.004836
positive regulation of T cell proliferation::GO:0042102	8	8.76E-05	0.004961
leukocyte aggregation::GO:0070486	20	9.28E-05	0.005121
cellular transition metal ion homeostasis::GO:0046916	9	0.0001023	0.005498
regulation of T cell activation::GO:0050863	15	0.0001227	0.006429

interleukin-1 beta secretion::GO:0050702	5	0.0001283	0.006561
transition metal ion homeostasis::GO:0055076	10	0.0001317	0.006574
purinergic receptor signaling pathway::GO:0035587	5	0.0001518	0.007401
positive regulation of lymphocyte proliferation::GO:0050671	9	0.0001556	0.007413
positive regulation of mononuclear cell proliferation::GO:0032946	9	0.0001664	0.007462
regulation of T cell proliferation::GO:0042129	10	0.0001673	0.007462
regulation of leukocyte cell-cell adhesion::GO:1903037	15	0.000167	0.007462
monosaccharide biosynthetic process::GO:0046364	8	0.0001825	0.00752
monocarboxylic acid metabolic process::GO:0032787	23	0.000183	0.00752
bone resorption::GO:0045453	6	0.000217	0.008749
interleukin-1 secretion::GO:0050701	5	0.0002424	0.009206
NADP metabolic process::GO:0006739	5	0.0002424	0.009206
regulation of tumor necrosis factor production::GO:0032680	8	0.000246	0.009206
regulation of homotypic cell-cell adhesion::GO:0034110	15	0.0002328	0.009206
interleukin-1 beta production::GO:0032611	6	0.0002701	0.009816
tumor necrosis factor production::GO:0032640	8	0.0002839	0.01008
ceramide metabolic process::GO:0006672	7	0.0004068	0.01354
regulation of lymphocyte proliferation::GO:0050670	11	0.0004152	0.01358
regulation of mononuclear cell proliferation::GO:0032944	11	0.0004345	0.01364
monovalent inorganic cation transport::GO:0015672	22	0.0004425	0.01364
nucleotide metabolic process::GO:0009117	24	0.0004396	0.01364
iron ion transport::GO:0006826	6	0.0004924	0.01496
cation transport::GO:0006812	33	0.0005674	0.01675
hexose biosynthetic process::GO:0019319	7	0.0006386	0.01809
cellular response to interferon-gamma::GO:0071346	9	0.000651	0.01819
negative regulation of tumor necrosis factor production::GO:0032720	5	0.0006834	0.01885
regulation of interleukin-1 beta production::GO:0032651	5	0.0009493	0.02456
activated T cell proliferation::GO:0050798	5	0.001053	0.02566
iron ion homeostasis::GO:0055072	7	0.001409	0.03109
cellular iron ion homeostasis::GO:0006879	6	0.001553	0.0339
hexose metabolic process::GO:0019318	12	0.001776	0.03447
Golgi vesicle transport::GO:0048193	12	0.001776	0.03447
metal ion transport::GO:0030001	26	0.001741	0.03447
carboxylic acid transport::GO:0046942	13	0.001836	0.03531
carboxylic acid biosynthetic process::GO:0046394	13	0.002259	0.04228
neuromuscular process controlling balance::GO:0050885	5	0.002605	0.04747
**Yellow module**			
mitochondrial translational elongation::GO:0070125	37	1.32E-42	2.05E-39
mitochondrial translational termination::GO:0070126	36	1.82E-40	1.41E-37
translational elongation::GO:0006414	41	2.90E-31	1.50E-28

translational termination::GO:0006415	38	3.75E-30	1.45E-27
translation::GO:0006412	56	6.10E-26	1.89E-23
peptide biosynthetic process::GO:0043043	57	7.69E-26	1.99E-23
amide biosynthetic process::GO:0043604	58	5.47E-24	1.21E-21
cellular protein complex disassembly::GO:0043624	39	7.42E-24	1.44E-21
protein complex disassembly::GO:0043241	39	1.69E-22	2.92E-20
nucleoside triphosphate metabolic process::GO:0009141	34	1.37E-20	2.13E-18
purine ribonucleoside monophosphate metabolic process::GO:0009167	33	4.68E-20	6.61E-18
purine nucleoside monophosphate metabolic process::GO:0009126	33	5.35E-20	6.93E-18
purine nucleoside triphosphate metabolic process::GO:0009144	32	7.03E-20	8.40E-18
purine ribonucleoside triphosphate metabolic process::GO:0009205	31	2.38E-19	2.46E-17
ribonucleoside monophosphate metabolic process::GO:0009161	33	2.22E-19	2.46E-17
ATP metabolic process::GO:0046034	30	3.40E-19	3.30E-17
ribonucleoside triphosphate metabolic process::GO:0009199	31	5.41E-19	4.94E-17
nucleoside monophosphate metabolic process::GO:0009123	33	8.58E-19	7.40E-17
ATP synthesis coupled electron transport::GO:0042773	19	1.23E-18	9.51E-17
mitochondrial ATP synthesis coupled electron transport::GO:0042775	19	1.23E-18	9.51E-17
mitochondrial electron transport, NADH to ubiquinone::GO:0006120	16	1.42E-17	1.05E-15
purine ribonucleoside metabolic process::GO:0046128	34	2.23E-17	1.58E-15
purine nucleoside metabolic process::GO:0042278	34	3.01E-17	2.04E-15
ribonucleoside metabolic process::GO:0009119	34	2.24E-16	1.45E-14
nucleotide metabolic process::GO:0009117	47	2.95E-16	1.83E-14
ribonucleotide metabolic process::GO:0009259	37	2.46E-13	1.47E-11
purine nucleotide metabolic process::GO:0006163	37	3.76E-13	2.16E-11
purine ribonucleotide metabolic process::GO:0009150	36	4.84E-13	2.68E-11
hydrogen ion transmembrane transport::GO:1902600	15	6.27E-10	3.36E-08
positive regulation of protein ubiquitination involved in ubiquitin-dependent protein catabolic process::GO:2000060	13	4.37E-09	2.26E-07
mitochondrial transport::GO:0006839	22	4.94E-09	2.48E-07
positive regulation of ubiquitin-protein ligase activity involved in regulation of mitotic cell cycle transition::GO:0051437	12	5.51E-09	2.67E-07
mitochondrial ATP synthesis coupled proton transport::GO:0042776	7	9.59E-09	4.51E-07
regulation of protein ubiquitination involved in ubiquitin-dependent protein catabolic process::GO:2000058	13	1.52E-08	6.92E-07

proton transport::GO:0015992	16	1.72E-08	7.64E-07
purine ribonucleoside monophosphate biosynthetic process::GO:0009168	11	2.84E-08	1.19E-06
purine nucleoside monophosphate biosynthetic process::GO:0009127	11	2.84E-08	1.19E-06
negative regulation of ubiquitin-protein ligase activity involved in mitotic cell cycle::GO:0051436	12	7.36E-08	3.01E-06
antigen processing and presentation of exogenous peptide antigen via MHC class I, TAP-dependent::GO:0002479	11	9.53E-08	3.79E-06
regulation of cell cycle arrest::GO:0071156	13	1.01E-07	3.91E-06
mitotic G1 DNA damage checkpoint::GO:0031571	11	1.10E-07	4.15E-06
G1 DNA damage checkpoint::GO:0044783	11	1.26E-07	4.55E-06
mitotic G1/S transition checkpoint::GO:0044819	11	1.26E-07	4.55E-06
energy coupled proton transport, down electrochemical gradient::GO:0015985	7	1.53E-07	5.17E-06
ATP synthesis coupled proton transport::GO:0015986	7	1.53E-07	5.17E-06
positive regulation of ubiquitin-protein transferase activity::GO:0051443	12	1.52E-07	5.17E-06
antigen processing and presentation of exogenous peptide antigen via MHC class I::GO:0042590	11	1.65E-07	5.33E-06
ribonucleoside monophosphate biosynthetic process::GO:0009156	11	1.65E-07	5.33E-06
negative regulation of ubiquitin-protein transferase activity::GO:0051444	12	1.71E-07	5.42E-06
ncRNA metabolic process::GO:0034660	25	2.00E-07	6.21E-06
regulation of ubiquitin-protein ligase activity involved in mitotic cell cycle::GO:0051439	12	2.15E-07	6.53E-06
negative regulation of protein ubiquitination::GO:0031397	14	2.19E-07	6.53E-06
DNA damage response, signal transduction by p53 class mediator resulting in cell cycle arrest::GO:0006977	10	2.69E-07	7.88E-06
signal transduction involved in mitotic G1 DNA damage checkpoint::GO:0072431	10	3.11E-07	8.63E-06
intracellular signal transduction involved in G1 DNA damage checkpoint::GO:1902400	10	3.11E-07	8.63E-06
protein ubiquitination involved in ubiquitin-dependent protein catabolic process::GO:0042787	15	3.10E-07	8.63E-06
signal transduction involved in mitotic cell cycle checkpoint::GO:0072413	10	4.13E-07	1.07E-05
signal transduction involved in mitotic DNA damage checkpoint::GO:1902402	10	4.13E-07	1.07E-05
signal transduction involved in mitotic DNA integrity checkpoint::GO:1902403	10	4.13E-07	1.07E-05
nucleoside monophosphate biosynthetic process::GO:0009124	11	3.99E-07	1.07E-05
RNA processing::GO:0006396	35	4.42E-07	1.12E-05

negative regulation of protein modification by small protein conjugation or removal::GO:1903321	14	5.16E-07	1.29E-05
signal transduction involved in DNA integrity checkpoint::GO:0072401	10	5.44E-07	1.32E-05
signal transduction involved in DNA damage checkpoint::GO:0072422	10	5.44E-07	1.32E-05
anaphase-promoting complex-dependent proteasomal ubiquitin-dependent protein catabolic process::GO:0031145	12	6.21E-07	1.46E-05
positive regulation of protein ubiquitination::GO:0031398	15	6.16E-07	1.46E-05
exonucleolytic nuclear-transcribed mRNA catabolic process involved in deadenylation-dependent decay::GO:0043928	7	6.39E-07	1.48E-05
ATP biosynthetic process::GO:0006754	8	7.02E-07	1.60E-05
nuclear-transcribed mRNA catabolic process, exonucleolytic::GO:0000291	7	1.32E-06	2.96E-05
regulation of mitotic cell cycle phase transition::GO:1901990	18	1.42E-06	3.14E-05
positive regulation of protein modification by small protein conjugation or removal::GO:1903322	15	1.54E-06	3.36E-05
proteasomal ubiquitin-independent protein catabolic process::GO:0010499	6	1.91E-06	4.11E-05
mitotic DNA damage checkpoint::GO:0044773	11	2.25E-06	4.66E-05
negative regulation of G1/S transition of mitotic cell cycle::GO:2000134	11	2.25E-06	4.66E-05
negative regulation of mitotic cell cycle phase transition::GO:1901991	14	2.19E-06	4.66E-05
negative regulation of cell cycle G1/S phase transition::GO:1902807	11	3.00E-06	6.13E-05
mitotic cell cycle checkpoint::GO:0007093	14	3.55E-06	7.16E-05
purine ribonucleoside triphosphate biosynthetic process::GO:0009206	8	3.83E-06	7.62E-05
mitotic DNA integrity checkpoint::GO:0044774	11	3.96E-06	7.79E-05
positive regulation of cell cycle arrest::GO:0071158	10	4.06E-06	7.88E-05
regulation of cell cycle phase transition::GO:1901987	18	4.21E-06	8.06E-05
purine nucleoside triphosphate biosynthetic process::GO:0009145	8	4.44E-06	8.31E-05
nucleoside triphosphate biosynthetic process::GO:0009142	9	4.41E-06	8.31E-05
negative regulation of cell cycle phase transition::GO:1901988	14	4.94E-06	9.12E-05
regulation of protein ubiquitination::GO:0031396	17	5.43E-06	9.92E-05
regulation of ubiquitin-protein transferase activity::GO:0051438	12	6.04E-06	0.0001091
DNA damage response, signal transduction by p53 class mediator::GO:0030330	11	6.17E-06	0.0001101
purine nucleoside biosynthetic process::GO:0042451	10	8.13E-06	0.0001418

purine ribonucleoside biosynthetic process::GO:0046129	10	8.13E-06	0.0001418
ribonucleoside triphosphate biosynthetic process::GO:0009201	8	8.88E-06	0.0001532
signal transduction by p53 class mediator::GO:0072331	13	1.00E-05	0.0001706
regulation of G1/S transition of mitotic cell cycle::GO:2000045	12	1.17E-05	0.0001979
positive regulation of proteolysis involved in cellular protein catabolic process::GO:1903052	13	1.21E-05	0.0002026
regulation of protein modification by small protein conjugation or removal::GO:1903320	17	1.37E-05	0.000226
carboxylic acid metabolic process::GO:0019752	37	1.46E-05	0.0002389
RNA splicing::GO:0008380	20	1.56E-05	0.000253
regulation of cell cycle G1/S phase transition::GO:1902806	12	2.16E-05	0.0003461
signal transduction in response to DNA damage::GO:0042770	11	2.19E-05	0.0003465
DNA damage checkpoint::GO:0000077	12	2.62E-05	0.0004116
positive regulation of cellular protein catabolic process::GO:1903364	13	2.67E-05	0.000415
ribonucleoside biosynthetic process::GO:0042455	10	2.75E-05	0.0004225
ribosome assembly::GO:0042255	7	2.96E-05	0.0004461
positive regulation of proteolysis::GO:0045862	19	2.95E-05	0.0004461
protein localization to mitochondrion::GO:0070585	13	3.36E-05	0.0005013
intracellular protein transmembrane import::GO:0044743	6	4.02E-05	0.0005952
G1/S transition of mitotic cell cycle::GO:0000082	15	4.59E-05	0.0006727
DNA integrity checkpoint::GO:0031570	12	4.84E-05	0.0007029
nucleoside biosynthetic process::GO:0009163	10	5.45E-05	0.0007834
cell cycle G1/S phase transition::GO:0044843	15	6.89E-05	0.0009811
porphyrin-containing compound biosynthetic process::GO:0006779	5	7.26E-05	0.001025
spliceosomal snRNP assembly::GO:0000387	6	8.61E-05	0.001205
NIK/NF-kappaB signaling::GO:0038061	9	8.70E-05	0.001206
RNA phosphodiester bond hydrolysis, endonucleolytic::GO:0090502	7	8.80E-05	0.001209
negative regulation of canonical Wnt signaling pathway::GO:0090090	11	9.30E-05	0.001256
RNA catabolic process::GO:0006401	14	9.22E-05	0.001256
proteasome-mediated ubiquitin-dependent protein catabolic process::GO:0043161	20	9.70E-05	0.001299
mitotic cell cycle phase transition::GO:0044772	21	0.0001123	0.001477
establishment of protein localization to mitochondrion::GO:0072655	12	0.0001174	0.001532
tetrapyrrole biosynthetic process::GO:0033014	5	0.0001285	0.001659
intracellular protein transmembrane transport::GO:0065002	6	0.0001292	0.001659

RNA splicing, via transesterification reactions::GO:0000375	15	0.0001463	0.001847
mRNA metabolic process::GO:0016071	25	0.0001456	0.001847
mRNA processing::GO:0006397	20	0.0001741	0.002163
nuclear-transcribed mRNA catabolic process, deadenylation-dependent decay::GO:0000288	7	0.0001804	0.002224
RNA phosphodiester bond hydrolysis::GO:0090501	9	0.0001927	0.002338
ncRNA processing::GO:0034470	15	0.0001925	0.002338
modification-dependent macromolecule catabolic process::GO:0043632	23	0.0002002	0.00241
proteasomal protein catabolic process::GO:0010498	20	0.0002018	0.002411
tumor necrosis factor-mediated signaling pathway::GO:0033209	10	0.0002138	0.002534
ATP hydrolysis coupled proton transport::GO:0015991	5	0.0002483	0.002899
energy coupled proton transmembrane transport, against electrochemical gradient::GO:0015988	5	0.0002483	0.002899
proteolysis involved in cellular protein catabolic process::GO:0051603	24	0.0002515	0.002915
stimulatory C-type lectin receptor signaling pathway::GO:0002223	9	0.0003441	0.003901
ubiquitin-dependent protein catabolic process::GO:0006511	22	0.0003572	0.00402
innate immune response activating cell surface receptor signaling pathway::GO:0002220	9	0.0004125	0.0046
modification-dependent protein catabolic process::GO:0019941	22	0.0004147	0.0046
mRNA splicing, via spliceosome::GO:0000398	14	0.0004361	0.00477
RNA splicing, via transesterification reactions with bulged adenosine as nucleophile::GO:0000377	14	0.0004361	0.00477
protein polyubiquitination::GO:0000209	12	0.0004795	0.005207
cellular protein catabolic process::GO:0044257	24	0.0005069	0.005467
tRNA metabolic process::GO:0006399	10	0.0005116	0.00548
positive regulation of canonical Wnt signaling pathway::GO:0090263	9	0.0006161	0.006553
protein ubiquitination::GO:0016567	26	0.0006987	0.007332
nuclear-transcribed mRNA catabolic process::GO:0000956	11	0.0007092	0.007392
insulin receptor signaling pathway::GO:0008286	15	0.001058	0.01074
mRNA catabolic process::GO:0006402	11	0.001215	0.01225
DNA-templated transcription, termination::GO:0006353	7	0.00128	0.01282
regulation of proteolysis involved in cellular protein catabolic process::GO:1903050	14	0.001327	0.01321
monovalent inorganic cation transport::GO:0015672	21	0.001713	0.01694
protein localization to organelle::GO:0033365	27	0.0018	0.0177
protein targeting::GO:0006605	24	0.001818	0.01776
cellular response to insulin stimulus::GO:0032869	16	0.001877	0.01822

cellular macromolecular complex assembly::GO:0034622	27	0.002286	0.02192
regulation of cellular protein catabolic process::GO:1903362	14	0.002326	0.02216
tRNA processing::GO:0008033	7	0.00261	0.02472
nucleotide biosynthetic process::GO:0009165	14	0.002673	0.02516
regulation of canonical Wnt signaling pathway::GO:0060828	11	0.002817	0.02619
protein modification by small protein conjugation::GO:0032446	26	0.003798	0.0349
aerobic respiration::GO:0009060	5	0.004799	0.04117
response to insulin::GO:0032868	16	0.005461	0.0466
regulation of proteolysis::GO:0030162	24	0.005888	0.04996
**Magenta module**			
ncRNA metabolic process::GO:0034660	25	2.68E-16	3.19E-13
rRNA metabolic process::GO:0016072	13	1.01E-10	6.02E-08
ncRNA processing::GO:0034470	16	1.87E-10	7.39E-08
rRNA processing::GO:0006364	12	9.58E-10	2.85E-07
RNA processing::GO:0006396	22	2.52E-08	6.00E-06
ribosome assembly::GO:0042255	6	1.50E-06	0.0002975
tRNA metabolic process::GO:0006399	9	1.78E-06	0.0003021
maturation of SSU-rRNA::GO:0030490	5	3.58E-06	0.0005326
tRNA aminoacylation for protein translation::GO:0006418	5	1.72E-05	0.002275
tRNA aminoacylation::GO:0043039	5	2.37E-05	0.00282
cellular response to cytokine stimulus::GO:0071345	16	3.30E-05	0.003569
translation::GO:0006412	13	0.000218	0.01851
amide biosynthetic process::GO:0043604	14	0.0003115	0.02313
peptide biosynthetic process::GO:0043043	13	0.0003308	0.02313
cytokine-mediated signaling pathway::GO:0019221	12	0.0006175	0.04079
**Lightyellow module**			
Category	genes in C	p value	fdr p value
B cell proliferation::GO:0042100	5	6.55E-07	0.0002339
positive regulation of cytosolic calcium ion concentration::GO:0	5	0.0001752	0.02085
regulation of cytosolic calcium ion concentration::GO:0051480	5	0.0002745	0.0245
cellular calcium ion homeostasis::GO:0006874	5	0.0008823	0.05393
calcium ion homeostasis::GO:0055074	5	0.0009906	0.05393
cellular divalent inorganic cation homeostasis::GO:0072503	5	0.001286	0.05393
divalent inorganic cation homeostasis::GO:0072507	5	0.001526	0.05393
