# Supplementary material for: Do sputum or circulating blood samples reflect the pulmonary transcriptomic differences of COPD patients? A multi-tissue transcriptomic network META-analysis
Source: Respir Res. 2019 Jan 8;20:5. doi: 10.1186/s12931-018-0965-y (PMC6325784; doi:10.1186/s12931-018-0965-y)
Supplement: Supplementary file 5 — Table S3. KEGG pathway enrichment in Yellow, Brown, and Magenta modules. (PDF 36 kb) [file 12931_2018_965_MOESM5_ESM.pdf]

Table S3

KEGG PATHWAY ID	# genes	p value	fdr p value
**Brown module**			
Lysosome::04142	31	2.13E-22	3.46E-20
Staphylococcus aureus infection::05150	11	2.11E-07	1.72E-05
Glycosaminoglycan degradation::00531	7	4.09E-07	2.22E-05
Other glycan degradation::00511	6	3.88E-06	0.0001278
Phagosome::04145	16	3.92E-06	0.0001278
Rheumatoid arthritis::05323	11	3.54E-05	0.0008994
Antigen processing and presentation::04612	10	3.86E-05	0.0008994
Glycosphingolipid biosynthesis - globo series::00603	4	0.000451	0.00919
Osteoclast differentiation::04380	11	0.0007548	0.01367
Amino sugar and nucleotide sugar metabolism::00520	6	0.001894	0.03088
Glutathione metabolism::00480	6	0.002344	0.03474
**Yellow module**			
Oxidative phosphorylation::00190	33	9.32E-26	8.58E-24
Parkinson's disease::05012	27	7.93E-19	3.65E-17
Huntington's disease::05016	30	8.53E-18	2.62E-16
Alzheimer's disease::05010	28	6.70E-17	1.54E-15
Metabolic pathways::01100	55	1.59E-08	2.92E-07
Proteasome::03050	8	6.98E-06	0.0001071
Spliceosome::03040	10	0.0007931	0.01042
RNA degradation::03018	7	0.00135	0.01552
**Magenta module**			
Ribosome biogenesis in eukaryotes::03008	10	2.22E-09	1.58E-07
