# Supplementary material for: Do sputum or circulating blood samples reflect the pulmonary transcriptomic differences of COPD patients? A multi-tissue transcriptomic network META-analysis
Source: Respir Res. 2019 Jan 8;20:5. doi: 10.1186/s12931-018-0965-y (PMC6325784; doi:10.1186/s12931-018-0965-y)
Supplement: Supplementary file 8 — Table S4. Core 60 genes in common between the yellow meta-analysis module and brown consensus module with the p-value <0.1 for association with FEV1 % predicted. (PDF 38 kb) [file 12931_2018_965_MOESM8_ESM.pdf]

Table S4

Gene.Symbol	gene_id	GS.set1.FEV1.PP	GS.set2.FEV1.PP	GS.set3.FEV1.PP	p.GS.set1.FEV1.PP	p.GS.set2.FEV1.PP	p.GS.set3.FEV1.PP
AHCY	191	-0.363535545	-0.308554066	0.271560599	0.001978975	0.000489224	0.002586856
ANAPC11	51529	-0.289324916	-0.181247194	0.338110458	0.015128771	0.043950744	0.000148873
ATIC	471	-0.335637022	-0.34314079	0.38351698	0.004504636	9.56E-05	1.41E-05
ATP5G2	517	-0.307792923	-0.188773944	0.421429995	0.009540084	0.035752994	1.48E-06
ATPAF1	64756	-0.212799851	-0.282817559	0.407195	0.076948582	0.001459259	3.57E-06
CCDC127	133957	-0.276753335	-0.207948988	0.288807402	0.020379167	0.020472963	0.001315157
CLNS1A	1207	-0.282235343	-0.322711549	0.386231362	0.017924401	0.000256708	1.21E-05
CLPP	8192	-0.28576765	-0.196432442	0.380231758	0.016480646	0.02877353	1.69E-05
CNPY2	10330	-0.293727485	-0.240039908	0.369675878	0.013588771	0.007246934	3.01E-05
COQ9	57017	-0.231763433	-0.275499624	0.37936641	0.053540952	0.00195539	1.78E-05
COX5B	1329	-0.235248836	-0.232865248	0.413403624	0.049948905	0.009249324	2.44E-06
DCTPP1	79077	-0.340287994	-0.194084135	0.360573885	0.003947285	0.03077859	4.85E-05
DPAGT1	1798	-0.39453782	-0.274155134	0.311104671	0.000725594	0.002061655	0.000514227
DPM2	8818	-0.336531423	-0.16242997	0.343950411	0.004392335	0.07148021	0.000112132
DUSP23	54935	-0.356638034	-0.262865502	0.269817133	0.002441933	0.003182315	0.002763447
ERGIC3	51614	-0.328086803	-0.155301562	0.415612829	0.0055586	0.085012199	2.13E-06
EXOSC7	23016	-0.300001475	-0.382387936	0.335385623	0.011628939	1.17E-05	0.000169597
FLAD1	80308	-0.351762076	-0.211688066	0.403825898	0.002825367	0.018265162	4.37E-06
GEMIN6	79833	-0.391926556	-0.207466206	0.376814904	0.000792594	0.020774139	2.04E-05
JAGN1	84522	-0.357821445	-0.250038724	0.352186216	0.002356199	0.005098458	7.45E-05
KRTCAP2	200185	-0.332542521	-0.166131138	0.337745069	0.004913053	0.065174595	0.000151508
LSM7	51690	-0.337415459	-0.179433601	0.294453298	0.004283781	0.046144761	0.001044111
MPV17L2	84769	-0.395700781	-0.322009426	0.247698932	0.000697446	0.000265251	0.006158335
MRPL11	65003	-0.213765267	-0.216745287	0.42814061	0.07558689	0.015608392	9.65E-07
MRPL36	64979	-0.344932474	-0.195264244	0.310416107	0.003452679	0.02975664	0.00052995
MRPL40	64976	-0.201146254	-0.383226517	0.430624498	0.094974431	1.12E-05	8.21E-07
MRPL52	122704	-0.322208353	-0.359978825	0.267498854	0.00652415	4.02E-05	0.003015082
MRPS11	64963	-0.336395203	-0.260503535	0.41627937	0.004409277	0.003476872	2.05E-06
MRPS12	6183	-0.399711938	-0.266844308	0.317116493	0.000607802	0.002736555	0.000394105
MRPS15	64960	-0.274986834	-0.316157159	0.388205041	0.021228907	0.00034739	1.08E-05
MRPS23	51649	-0.245632344	-0.336772944	0.353276773	0.040401475	0.000131036	7.05E-05

**MRPS33**	51650	-0.262007056	-0.240765446	0.425395476	0.028446782	0.007067599	1.15E-06
**NDUFA3**	4696	-0.329418597	-0.249340565	0.416671858	0.00535823	0.005227514	2.00E-06
**NDUFA8**	4702	-0.203178329	-0.197557126	0.367146064	0.091613114	0.027853274	3.44E-05
**NDUFB1**	4707	-0.202813577	-0.16410461	0.402625424	0.092209477	0.068568546	4.69E-06
**NDUFB11**	54539	-0.261957602	-0.219669795	0.222801543	0.02847781	0.014230725	0.014036189
**NDUFB2**	4708	-0.316125838	-0.215836011	0.321710135	0.007675534	0.016059706	0.000320396
**NHP2**	55651	-0.305054384	-0.222274022	0.3583314	0.0102336	0.013094369	5.45E-05
**NSDHL**	50814	-0.294373201	-0.229819178	0.299407607	0.013374697	0.010237419	0.000849402
**NUDT22**	84304	-0.403377806	-0.184481494	0.361311302	0.000535181	0.040253919	4.67E-05
**PGLS**	25796	-0.231913432	-0.289092647	0.274563571	0.053382137	0.00112827	0.002306431
**POLR3K**	51728	-0.200593055	-0.167107857	0.467756045	0.095906021	0.063588392	6.32E-08
**PSMB3**	5691	-0.267487809	-0.207292963	0.220582892	0.025180021	0.02088314	0.015046756
**RNASEH2C**	84153	-0.251924233	-0.269218122	0.246521139	0.03539069	0.002498249	0.00641485
**RPL39L**	116832	-0.314393204	-0.264187349	0.192697677	0.00803451	0.003027457	0.034212285
**RWDD1**	51389	-0.248070377	-0.234299135	0.357548991	0.038394192	0.00881402	5.67E-05
**SCAND1**	51282	-0.363620866	-0.268705389	0.178879717	0.001973779	0.002548074	0.049632649
**SF3B5**	83443	-0.392685268	-0.23490303	0.306605407	0.000772571	0.008636165	0.000625252
**SFXN4**	119559	-0.350038457	-0.186902998	0.343819922	0.00297325	0.037659796	0.000112851
**SNRPD2**	6633	-0.262388853	-0.264534341	0.309674877	0.028208192	0.002987948	0.000547369
**SUMO3**	6612	-0.288647525	-0.347215056	0.254835361	0.01537858	7.79E-05	0.004790106
**SURF1**	6834	-0.218319241	-0.259184019	0.328470898	0.069420833	0.003651889	0.000234814
**TMED3**	23423	-0.31453099	-0.307703439	0.388483168	0.008005434	0.00050804	1.07E-05
**TMEM147**	10430	-0.2634419	-0.26940802	0.312695392	0.027558849	0.00248002	0.000479532
**TOMM22**	56993	-0.33447971	-0.255358916	0.379158289	0.004653718	0.004204823	1.80E-05
**TSFM**	10102	-0.406434093	-0.314988632	0.370038734	0.000480777	0.000366376	2.95E-05
**UQCRC1**	7384	-0.322615833	-0.198137643	0.196245276	0.006452753	0.027388162	0.030981185
**UQCRQ**	27089	-0.234590523	-0.272886512	0.394043655	0.050611795	0.002166694	7.76E-06
**UXT**	8409	-0.40125688	-0.328753649	0.27897266	0.000576169	0.00019305	0.001944337
**VKORC1**	79001	-0.224749052	-0.234045939	0.314440143	0.061409467	0.008889546	0.000443965
