# Supplementary material for: Do sputum or circulating blood samples reflect the pulmonary transcriptomic differences of COPD patients? A multi-tissue transcriptomic network META-analysis
Source: Respir Res. 2019 Jan 8;20:5. doi: 10.1186/s12931-018-0965-y (PMC6325784; doi:10.1186/s12931-018-0965-y)
Supplement: Supplementary file 9 — Table S5. Gene Ontology enrichment for the core 60 genes. (PDF 33 kb) [file 12931_2018_965_MOESM9_ESM.pdf]

Table S5

Category	genes.in.Category	p.value	fdr.p.value
mitochondrial translation::GO:0032543	11	1.47E-13	3.51E-11
mitochondrial translational elongation::GO:0070125	10	1.52E-13	3.51E-11
mitochondrial gene expression::GO:0140053	11	5.35E-13	8.23E-11
mitochondrial ATP synthesis coupled electron transport::GO:0042775	9	5.83E-12	5.92E-10
ATP synthesis coupled electron transport::GO:0042773	9	6.51E-12	5.92E-10
translational elongation::GO:0006414	10	8.78E-12	5.92E-10
mitochondrial translational termination::GO:0070126	9	8.97E-12	5.92E-10
translational termination::GO:0006415	9	3.47E-11	2.01E-09
purine ribonucleoside monophosphate metabolic process::GO:0009167	12	2.32E-10	1.11E-08
purine nucleoside monophosphate metabolic process::GO:0009126	12	2.41E-10	1.11E-08
ribonucleoside monophosphate metabolic process::GO:0009161	12	3.59E-10	1.38E-08
nucleoside triphosphate metabolic process::GO:0009141	12	3.59E-10	1.38E-08
nucleoside monophosphate metabolic process::GO:0009123	12	6.04E-10	2.15E-08
ATP metabolic process::GO:0046034	11	7.11E-10	2.35E-08
purine ribonucleoside triphosphate metabolic process::GO:0009205	11	2.19E-09	6.74E-08
ribonucleoside triphosphate metabolic process::GO:0009199	11	2.61E-09	7.53E-08
purine nucleoside triphosphate metabolic process::GO:0009144	11	2.80E-09	7.60E-08
nucleotide metabolic process::GO:0009117	15	5.24E-09	1.35E-07
mitochondrial electron transport, NADH to ubiquinone::GO:0006120	6	7.20E-09	1.75E-07
cellular protein complex disassembly::GO:0043624	9	1.34E-08	3.10E-07
protein complex disassembly::GO:0043241	9	2.03E-07	4.40E-06
purine ribonucleotide metabolic process::GO:0009150	12	2.18E-07	4.40E-06
amide biosynthetic process::GO:0043604	15	2.19E-07	4.40E-06
ribonucleotide metabolic process::GO:0009259	12	2.88E-07	5.41E-06
purine nucleotide metabolic process::GO:0006163	12	2.93E-07	5.41E-06
translation::GO:0006412	14	3.43E-07	6.00E-06
mitochondrial respiratory chain complex assembly::GO:0033108	6	3.51E-07	6.00E-06
peptide biosynthetic process::GO:0043043	14	5.00E-07	8.25E-06
cellular macromolecular complex assembly::GO:0034622	13	1.97E-05	0.0002929
cellular protein complex assembly::GO:0043623	7	0.002906	0.02401
mRNA metabolic process::GO:0016071	8	0.004264	0.02985
